# TGF-alpha/EGFR signalling mediates retinoic acid-induced lung repair

**DOI:** 10.1038/s41536-026-00492-9

**Published:** 2026-07-04

**Authors:** Sek-Shir Cheong, Chunyu Yan, Róisín Mongey, David Chambers, Mark Griffiths, Matthew Hind, Charlotte H. Dean

**Affiliations:** 1 National Heart and Lung Institute (NHLI), Imperial College London, London, UK; 2 Wolfson Centre for Age Related Diseases, King’s College London, London, UK; 3 Peri-Operative Medicine Department, St Bartholomew’s Hospital, London, UK; 4 National Institute for Health Research (NIHR) Respiratory Biomedical Research Unit at the Royal Brompton & Harefield NHS Foundation Trust, London, UK

**Keywords:** Cancer, Cell biology, Diseases, Molecular biology

## Abstract

Lung repair involves coordination of multicellular processes, including endothelial angiogenesis and epithelial repopulation. Retinoic acid (RA) signalling is crucial for lung development, homeostasis, and repair; however, the mechanisms through which RA drives repair are still unknown. It has previously been shown that RA has no direct effects on the repair of alveolar epithelium, yet in animal studies, RA induces alveolar regeneration. Here we show that RA induces endothelial angiogenesis, which is associated with enhanced paracrine signalling to alveolar epithelial cells. Transcriptomic profiling of RA-treated HPMECs undergoing angiogenesis revealed enrichment of wound healing pathways, and subsequent in silico analysis identified several candidate mediators of endothelial-epithelium crosstalk. Scratch assays demonstrated that of these candidates, only TGFα promoted wound healing in alveolar epithelial A549 and primary human alveolar type 2 (hAT2) cells. Mechanistically, TGFα was associated with increased epithelial cell migration and activation of EGFR signalling, without detectable effects on proliferation or apoptosis. Our findings indicate that RA-induced endothelial angiogenesis promotes epithelial EGFR signalling through paracrine mediators, including TGFα. This study highlights the importance of endothelial–epithelial interactions in lung repair and provides insight into pathways that may be targeted to enhance alveolar regeneration.

**Figure:**
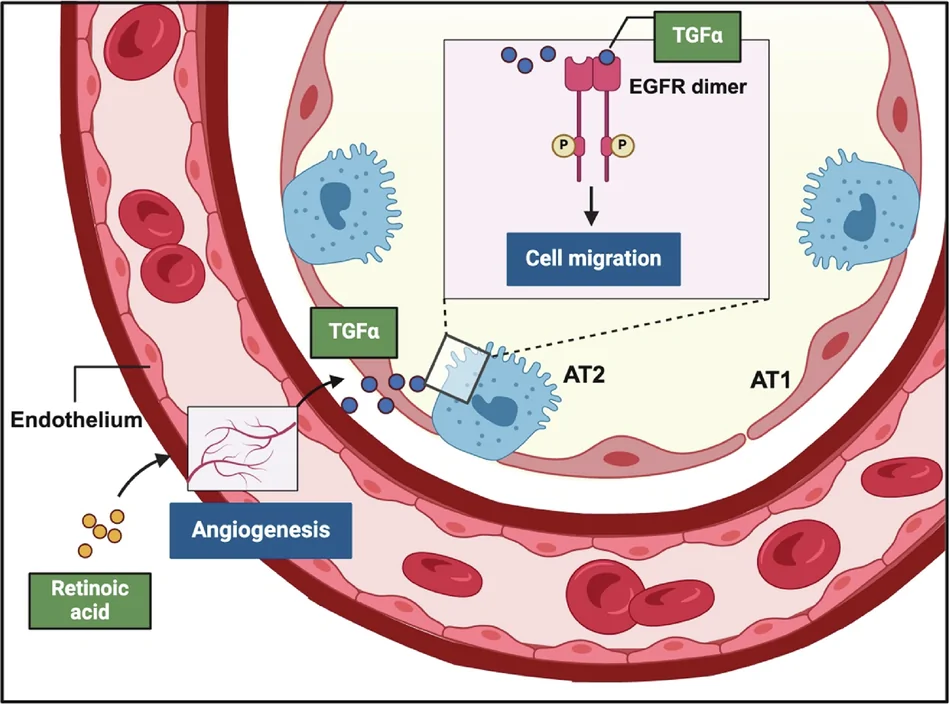

## Introduction

A common denominator in many lung diseases is insufficient functional alveolar surface area for gas exchange. The root cause of this may differ, for example in emphysema, a pathology associated with chronic obstructive pulmonary disease (COPD), the alveoli are destroyed due to repeated insults to the lungs causing tissue destruction. In the case of bronchopulmonary dysplasia, a disease of prematurity, infants fail to form an appropriate number of alveoli, leading to compromised gas exchange requiring oxygen support and lifelong increased susceptibility to other lung diseases. Currently, there are no treatments capable of restoring damaged lung parenchyma, but one promising avenue through which this might be achieved, is to develop a regenerative medicine treatment to repair or (re)grow alveoli^1,2^.

The lungs possess significant ability to regenerate and repair following injury, and we are beginning to understand the underlying cellular mechanisms governing these processes^3^. For example, there are populations of tissue stem or progenitor cells that reside at different anatomical locations in the lungs, which can become activated upon lung injury and contribute to regenerating the tissue^4,5^. In addition, other cell behaviours such as cell migration, proliferation, and cell plasticity contribute to the repair of lung tissue^6^. Pharmacological-based modification of alveolar repair has begun to show real therapeutic potential for treating parenchymal lung diseases^7^.

One possible approach to develop a regenerative medicine treatment is to identify the molecular signals that drive endogenous lung repair/regeneration and harness these to repair damaged lung tissue. However, this requires detailed knowledge about the endogenous pro-repair pathways. Emerging lung pro-repair factors include Wingless-type MMTV integration site family (Wnts), fibroblast growth factors (FGFs), and sonic hedgehog growth factors^8–10^. In addition, the vitamin A metabolite, retinoic acid (RA), plays a critical role in lung development and alveolar regeneration^11,12^.

To date, research on alveolar repair and regeneration has largely focused on the epithelial population, with comparatively less attention given to the capillary endothelium, despite its equally critical role in gas exchange^13^. RA is a well-known pro-regenerative factor across species, and studies in animal models demonstrated its ability to drive alveolar regeneration^14^. Human studies identified an imbalance between synthesis and breakdown of RA metabolism in emphysematous lungs, and RA signalling was activated in the microvascular endothelium, which stimulates angiogenesis but does not directly induce epithelial repair^15^. In addition, Ding and colleagues showed that endothelial-derived angiocrine signals induce and sustain alveolar regeneration^16^. Building on these research findings, we hypothesised that factors produced by the microvascular endothelium during RA-induced angiogenesis may act as endogenous mediators that influence alveolar epithelial repair through paracrine signalling. To test this hypothesis, we conducted microarray analysis of RA-treated lung microvascular endothelial cells undergoing angiogenesis and identified several candidate genes encoding intercellular signalling proteins. We subsequently tested the pro-repair capabilities of these proteins in vitro and found that transforming growth factor-α (TGFα) promoted repair responses in human alveolar epithelial cells, in association with activation of epidermal growth factor receptor (EGFR) signalling. TGFα, one of seven known EGFR ligands, has previously been shown to be important for alveologenesis^17^. These key mechanistic findings provide insight into potential mechanisms through which RA-regulated endothelial activity may influence epithelial repair and highlight pathways that could be explored for regenerative therapeutic strategies in chronic lung disease.

## Results

### Increased RA signalling activity following acid injury in mouse precision-cut lung slices (PCLS)

**Fig. 1:**
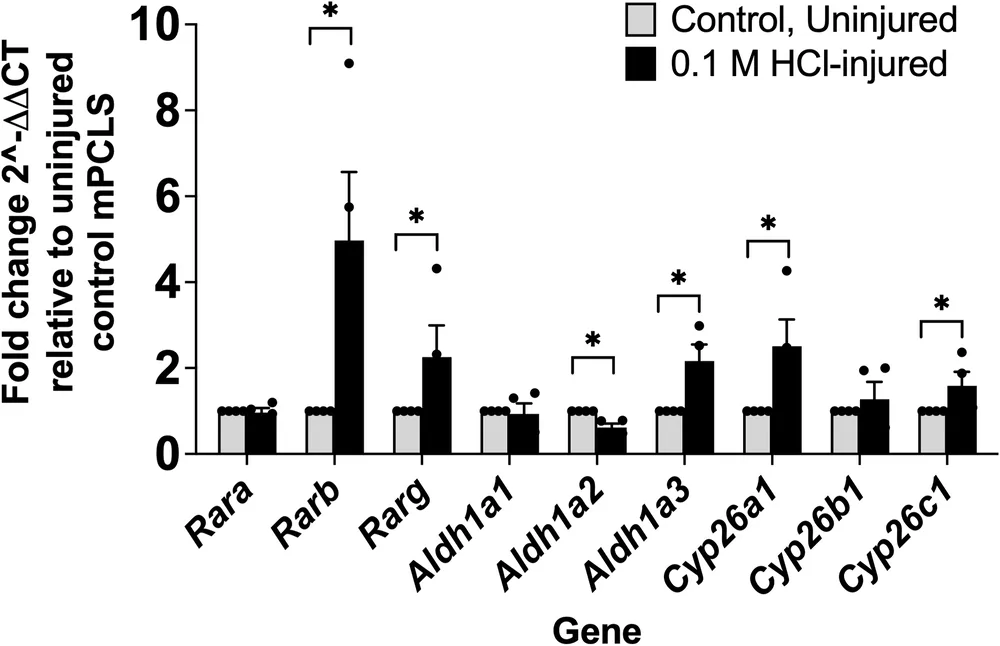
Increased RA signalling activity following acid injury in mouse PCLS. Histogram showing transcript levels of genes associated with the RA signalling pathway, including RA receptors (*Rara, Rarb*, and *Rarg*), RA synthesis genes (*Aldh1a1, Aldh1a2*, and *Aldh1a3*), and RA degradation genes (*Cyp26a1*, *Cyp26b1*, and *Cyp26c1*), assessed 48 h after injuring mouse PCLS with 0.1 M HCl. Gene expression was normalised to the housekeeping gene *B2m* and expressed as fold change relative to uninjured control PCLS. *n* = 4 mice; Mann–Whitney *U*-test, *p < 0.05.

To explore the role of the RA signalling pathway in lung repair, we generated mouse PCLS and conducted quantitative real-time PCR (qRT-PCR) to assess the expression of RA pathway components in uninjured controls versus acid-injured PCLS. Acid injury was induced by exposure to 0.1 M HCl for 1 min, and mRNA levels of RA receptors (*Rara, Rarb*, and *Rarg*), RA synthesis genes (*Aldh1a1, Aldh1a2*, and *Aldh1a3*), and RA degradation genes (*Cyp26a1*, *Cyp26b1*, and *Cyp26c1*) were quantified 48 h post-injury. As shown in Fig. 1, *Rarb* and *Rarg* transcript levels were significantly increased by 4.9-fold (*p* = 0.029) and 2.3-fold (*p* = 0.029), respectively, following acid injury of mouse PCLS. The RA synthesis gene *Aldh1a3* was also significantly upregulated, showing a 2.2-fold increase (*p* = 0.029), whereas *Aldh1a2* expression decreased to 0.6-fold (*p* = 0.029). Additionally, the RA degradation genes *Cyp26a1* and *Cyp26c1* showed significant upregulation with increases of 2.5-fold (*p* = 0.029) and 1.6-fold (*p* = 0.029), respectively, compared to uninjured control PCLS. No significant changes were observed for *Rara*, *Aldh1a1*, or *Cyp26b1*.

Collectively, these data suggest an increased RA signalling activity following acid injury, highlighting a role for RA signalling in the early stages of lung repair.

### RA stimulates angiogenesis and wound healing in endothelial cells, but not in alveolar epithelial cells

**Fig. 2:**
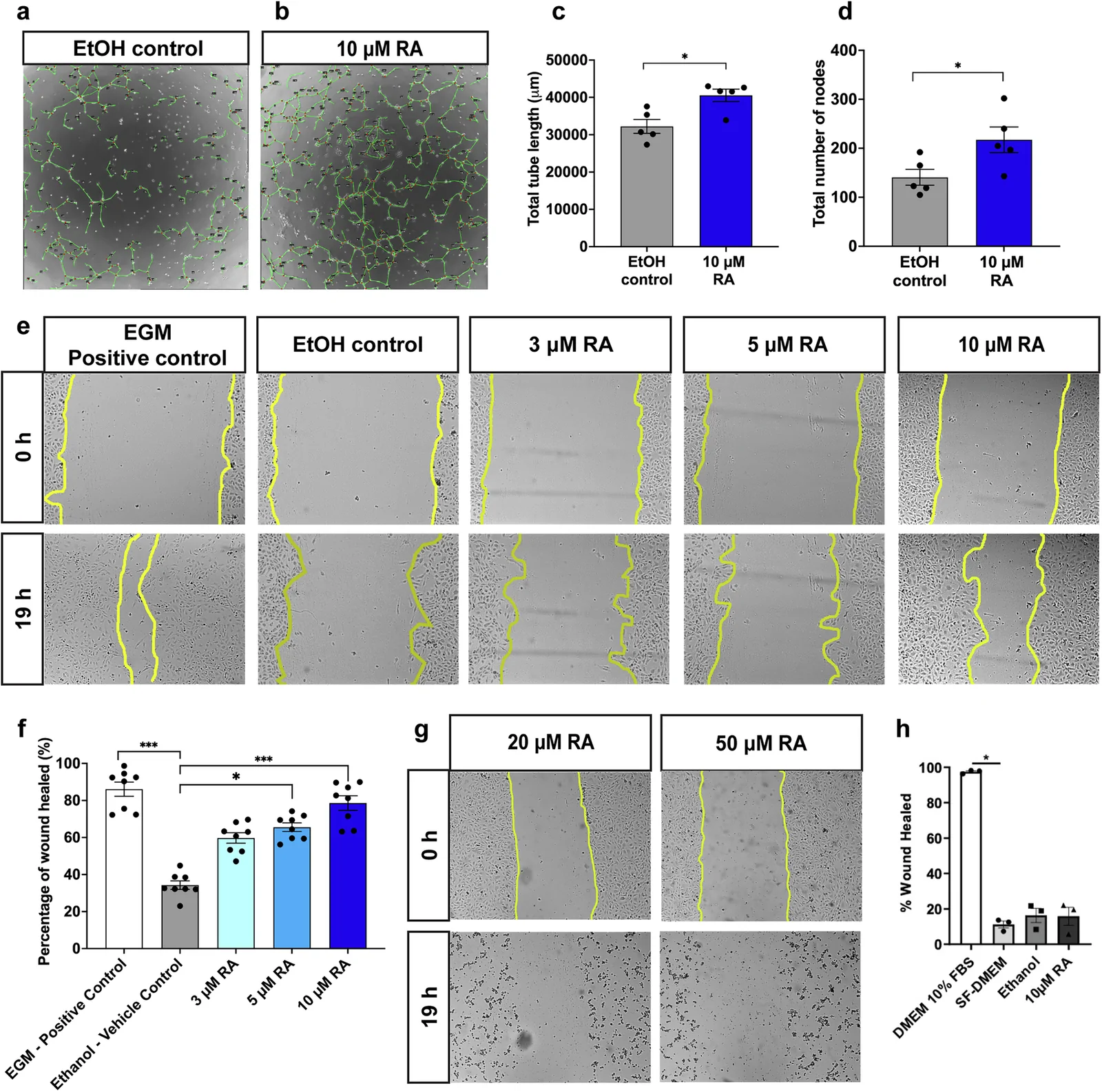
RA enhances wound healing and angiogenesis in endothelial cells but does not affect alveolar epithelial A549 cells. **a**, **b** Representative images showing endothelial tube-like structures. Tube lengths are depicted as green lines, and nodes are indicated by red dots. **c**, **d** Quantification of total tube length (**c**) and number of nodes (**d**) per field of view in HPMECs treated with either EtOH control or 10 μM RA at 90 min post-treatment. *n* = 5 independent experiments; each experiment was run in triplicate; each dot represents the mean value per experiment. Mann–Whitney *U*-test, *p < 0.05. **e** Representative images of HUVEC wound closure following treatment with 3, 5, and 10 μM RA, compared to EGM positive control and EBM + 0.1% EtOH vehicle controls, at 0 and 19 h post-scratch. Wound edges are marked with yellow lines. **f** Percentage of wound healed measured at 19 h post-scratch in HUVECs. *n* = 3 indepe*n*dent experiments; two or three technical replicates per experiment; each dot represents a technical replicate. Data were presented as mean ± SEM; Kruskal–Wallis with Dunn’s multiple comparisons test, *p < 0.05, ***p < 0.001. **g** Images of HUVECs treated with 20 and 50 μM RA at 0 and 19 h post-scratch. **h** Histogram showing the percentage of wound healing in A549 cells treated with 10 μM RA compared to DMEM + 10% FBS positive control and serum-free DMEM + 0.1% EtOH vehicle controls at 0 and 19 h post-scratch. *n* = 3 independent experiments; each dot represents the mean percentage of wound healed per experiment. Data were presented as mean ± SEM; Kruskal–Wallis with Dunn’s multiple comparisons test, *p < 0.05.

Our previous work demonstrated that RA induced angiogenesis in human pulmonary microvascular endothelial cells (HPMECs)^15^. As a prelude to microarray experiments, we replicated the experiment. Consistent with previous findings, we found that tube length, averaging 40,546 μm per field of view with RA treatment, compared to 32,225 μm in the 0.1% vehicle ethanol (EtOH) control (*p* = 0.0317) (Fig. 2a–c). Furthermore, the total number of nodes per field of view increased from 141 in the control to 217 in RA-treated HPMECs (*p* = 0.0317) (Fig. 2d).

To assess whether RA also modulates wound healing capacity in other endothelial cell types, scratch wound assays were performed on human umbilical vein endothelial cells (HUVECs) treated with varying concentrations of RA. Compared to vehicle EtOH-treated controls, RA significantly enhanced wound healing in a dose-dependent manner. The most pronounced effect was observed at 10 μM RA, resulting in an 80.1% wound healing compared to 38.8% in the control (*p* = 0.0002). Treatment with 5 μM RA led to 67.2% wound closure (*p* = 0.0341), while 3 μM RA increased closure to 61.0%, though this was not statistically significant. HUVECs cultured in complete endothelial growth media (EGM) with 3% foetal calf serum (FCS), serving as a positive control, exhibited high wound healing capacity (86.1%) (Fig. 2e, f). However, higher RA concentrations (20 and 50 μM) were toxic, leading to cell death after 19 hours of incubation (Fig. 2g).

We also confirmed that consistent with previous findings^15^, direct treatment of the alveolar epithelial cell line A549 with RA did not induce wound healing (Fig. 2h). A549 cells cultured in DMEM supplemented with 10% foetal bovine serum (FBS) served as a positive control, demonstrating nearly complete wound closure after 19 h (Fig. 2h).

### Transcriptomic profiling of RA-induced responses in HPMECs

**Fig. 3:**
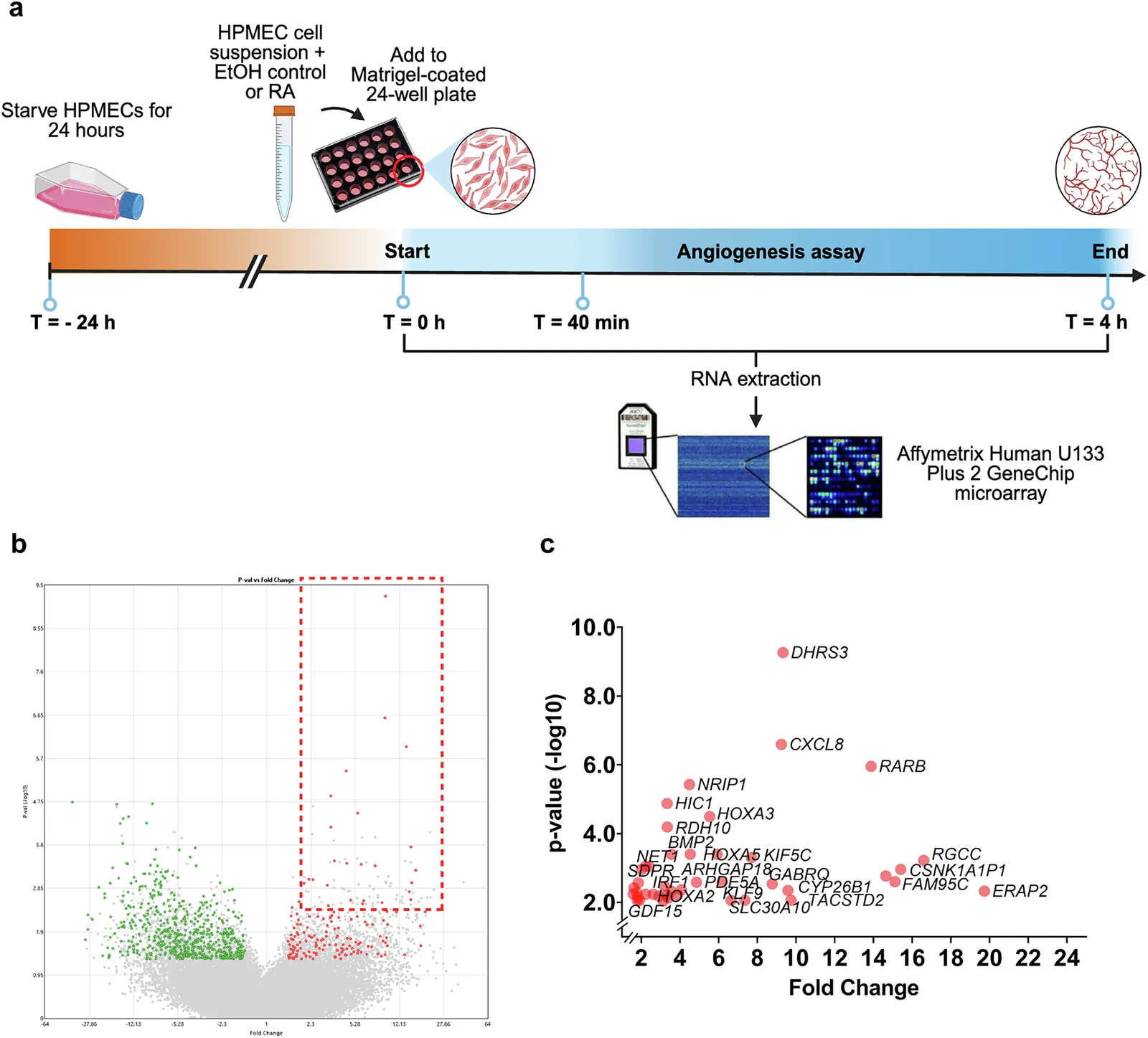
HPMECs respond to exogenous RA at 4 h post-treatment. **a** Schematic diagram of the HPMEC angiogenesis assay and the timeline for RNA extraction for microarray profiling using Affymetrix Human U133 Plus 2 GeneChip microarrays. Total RNA was extracted from HPMECs seeded on Matrigel at 0 h, 40 min, and 4 h after treatment. *n* = 4 independent experiments; each RNA sample was produced by pooling three technical replicates. The diagram was created in BioRender. **b** Volcano plot of differentially expressed genes (DEGs) in HPMECs treated with 10 μM RA versus EtOH control at 4 h. Upregulated genes are shown in red, downregulated genes in green. **c** Extrapolated volcano plot highlighting upregulation of key RA signalling components, including *RARB, DHRS3*, *NRIP1*, and *RDH10*, at 4 h after RA treatment.

To investigate the mechanisms underlying RA-induced angiogenesis in HPMECs, transcriptomic profiling was performed to examine gene expression changes during this process. Time-lapse imaging of angiogenesis was first conducted to determine critical time points for further analysis. As shown in Supplementary Video S1, RA-treated HPMECs rapidly migrated and reorganised into tube-like structures within 40 minutes of seeding on Matrigel, whereas vehicle-treated (EtOH) controls remained randomly distributed at the same time point (Video S2). By 60 min, clear differences in tube formation were evident between control and RA-treated cells (Videos S1 and S2). By 200 min, extensive tube networks had formed in RA-treated HPMECs. Based on these observations, RNA was extracted at 40 min, when tube initiation was first apparent, and at 4 h, when significant networks were evident, for whole-genome expression analysis comparing EtOH control versus RA-treated HPMECs (Fig. 3a).

Microarray analysis identified 962 differentially expressed genes (DEGs) at 40 min and 545 DEGs at 4 h post-treatment between control and RA-treated HPMECs [fold change >1.5 or <−1.5, false detection rate (FDR)-adjusted *p* value <0.05] (Supplementary Tables S1 and S2). Notably, volcano plot analysis highlighted key components of the RA signalling pathway, including *RARB* (retinoic acid receptor beta), *DHRS3* (dehydrogenase/reductase 3), *NRIP1* (nuclear receptor interacting protein 1), and *RDH10* (retinol dehydrogenase 10), among the most upregulated genes at 4 h post-RA treatment (Fig. 3b, c). In contrast, none of the top genes upregulated at 40 min were related to RA signalling (Fig. S1).

RA is the primary ligand for RAR-RXR heterodimers, which bind to retinoic acid response elements (RAREs) to initiate transcription of RA target genes^18^. To further investigate the response of HPMECs to RA, the top 15 DEGs at both the 40-minute and 4-h time points were analysed to determine whether these genes contained a RARE motif (Supplementary Tables S3 and S4). In silico prediction of RARE DR5 sequences was performed using oPOSSUM3, and results were cross-referenced with previously published RARE-containing genes^19,20^. Interestingly, the top 15 upregulated genes at 4 h showed significant enrichment for RARE-containing genes (47%; 7 out of 15 genes), including *DHRS3, RARB, NRIP1, HIC1, HOXA3, HOXA5*, and *ARHGAP18* (Table S3). In contrast, none of the top 15 upregulated genes at 40 min contained RARE sequences (Table S4). These findings strongly suggest that a cellular response to exogenous RA has occurred by 4 h post-treatment, leading us to focus our subsequent gene enrichment and functional analyses on the 4-h dataset.

Previous studies have shown that RA induces the upregulation of vascular endothelial growth factor A (*VEGFA*)^15^. Consistent with this, our findings showed increased *VEGFA* expression at 40 min and upregulation of vascular endothelial growth factor receptor 1 (*VEGFR1* or *FLT1*) at 4 h post-RA treatment. No changes were found in other genes involved in VEGF signalling, including *VEGFB*, *VEGFC, FLT4 (VEGFR3), KDR (VEGFR2)*, neuropilin-1 (*NRP1*), and neuropilin-2 (*NRP2*) (Fig. S2).

### Enriched GO terms and pathways in RA-treated HPMECs

**Fig. 4:**
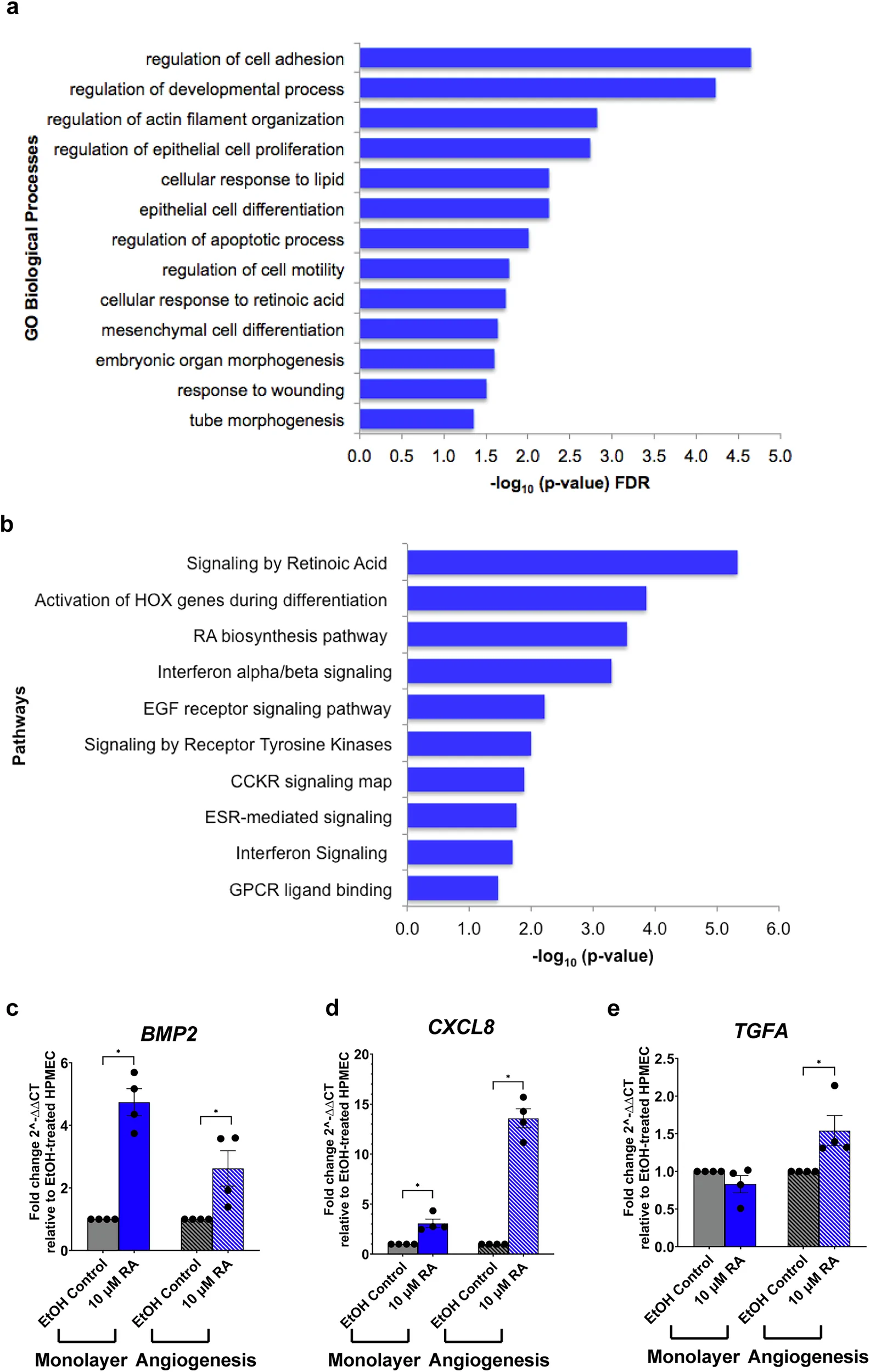
Gene set enrichment analysis and validation of expression change in candidate genes. **a** Histogram showing Gene Ontology (GO) biological process terms enriched in upregulated genes in RA-treated HPMECs, analysed using the Cytoscape STRING enrichment tool. **b** Over-represented pathways in RA-treated HPMECs identified by PANTHER and Reactome pathway analyses. Biological processes and pathways are ranked by statistical significance. **c**–**e** Transcript levels of *BMP2* (**c**), *CXCL8* (**d**), and *TGFA* (**e**) in EtOH control or 10 μM RA-treated HPMEC monolayers or HPMECs undergoing angiogenesis. *n* = 4 independent experiments; each experiment was run in triplicate. Data were presented as mean ± SEM; Mann–Whitney *U-*test, *p < 0.05.

Since cellular responses to RA have occurred by 4 h post-treatment (Fig. 3b, c and Table S3), we performed gene set enrichment analysis (GSEA) on DEGs from this time point using Panther and Cytoscape. Several key biological processes were significantly enriched in RA-treated HPMECs. The most prominent processes included regulation of cell adhesion, developmental processes, and actin filament organisation, all highly significant after FDR adjustment. Additional enriched processes, including epithelial cell proliferation, differentiation, cell motility, response to wounding, and tube morphogenesis, pointed to a robust cellular response to RA (Fig. 4a).

Pathway enrichment analysis further confirmed activation of RA signalling in RA-treated HPMECs. Pathways directly related to RA, including retinoic acid signalling and RA biosynthesis, were significantly enriched at 4 h post-treatment (Fig. 4b). Additionally, pathways such as interferon signalling and CCKR signalling suggest that RA also modulates immune responses. Notably, the EGF receptor (EGFR) signalling pathway, which has been implicated in lung development^21^ and various chronic pulmonary diseases, including lung fibrosis, COPD, asthma, and lung cancer^22–24^, as well as lung repair^25^, was also significantly enriched.

### Identification of candidate paracrine mediators upregulated in RA-treated HPMECs

Lung repair requires coordinated crosstalk among alveolar cell types^26^. Since RA promoted angiogenesis and wound healing in endothelial cells but had no direct effect on alveolar epithelial cells (Fig. 2), we hypothesised that RA signalling may induce the expression of pro-repair paracrine factors in endothelial cells that act on other alveolar cell populations. To identify candidate paracrine mediators, the 545 DEGs detected at 4 hours post-RA treatment were subjected to Panther “Protein classification” analysis. This revealed 14 candidate genes encoding intercellular signalling proteins, of which eight were upregulated, including *CXCL8* (C-X-C motif chemokine ligand 8)*, TGFA* (transforming growth factor alpha)*, BMP2* (bone morphogenetic protein 2)*, FGF20* (fibroblast growth factor 20)*, WNT9A* (Wnt family member 9a)*, GDF15* (growth differentiation factor 15)*, EFNB1* (Ephrin B1), and *ADM* (adrenomedullin) (Table 1). Three genes, *CXCL8, TGFA*, and *BMP2*, were selected for further analysis due to their known association with lung diseases and relevant biological functions^27–29^. Notably, TGFα, encoded by *TGFA*, is a ligand for the EGFR signalling pathway, which was one of the top-enriched pathways in RA-treated HPMECs (Fig. 4b).

**Table 1:** Identification of 14 genes encoding intercellular signalling proteins

Gene symbol	Protein class	Mapped to signalling pathway	Fold Change	FDR *P* value
*CXCL8*	Chemokine	CCKR signalling map, Interleukin signalling pathway, Inflammation mediated by chemokine and cytokine signalling pathway	9.24	2.56E-07
*TGFA*	Growth factor	EGF receptor signalling pathway	4.56	0.0176
*BMP2*	Growth factor	TGF-beta signalling pathway, Gonadotropin-releasing hormone receptor pathway	3.57	0.0004
*FGF20*	Growth factor	FGF signalling pathway	2.88	0.0184
*WNT9A*	Intercellular signal molecule	Wnt signalling pathway, cadherin signalling pathway	2.02	0.0376
*GDF15*	Growth factor	TGF-beta signalling pathway	1.79	0.0087
*EFNB1*	Membrane-bound signalling molecule	Angiogenesis	1.7	0.0286
*ADM*	Peptide hormone	-	1.58	0.0303
*CCL23*	Cytokine	-	−9.12	0.0188
*WNT5A*	Intercellular signal molecule	Angiogenesis, Wnt signalling pathway, cadherin signalling pathway	−8.18	0.0167
*PDYN*	Neuropeptide	Enkephalin release, Opioid prodynorphin pathway, Opioid proenkephalin pathway	−4.05	0.0241
*PTN*	Growth factor		−3.34	0.0473
*FGF5*	Growth factor	FGF signalling pathway	−2.22	0.0129
*FGF8*	Growth factor	FGF signalling pathway	−2.11	0.0484

To determine whether RA-induced gene expression is dependent on endothelial state, we compared transcriptional responses in RA-treated HPMECs cultured as monolayers versus undergoing angiogenesis. qRT-PCR analysis revealed that *BMP2* and *CXCL8* were consistently upregulated in response to RA under both conditions (*p* < 0.05), indicating that these genes are directly induced by RA signalling (Fig. 4c, d). In contrast, *TGFA* expression was not significantly altered in RA-treated HPMEC monolayers (0.83-fold relative to EtOH-treated HPMEC monolayer, *p* = 0.314), but was significantly upregulated in RA-treated HPMECs undergoing angiogenesis (1.54-fold increase, *p* = 0.029; Fig. 4e). *HOXA3* and *HOXA5*, were also upregulated in RA-treated HPMEC monolayers, further supporting that they are direct targets of RA signalling (Fig. S3).

### TGFα induces wound healing and cell migration in alveolar epithelial cells

**Fig. 5:**
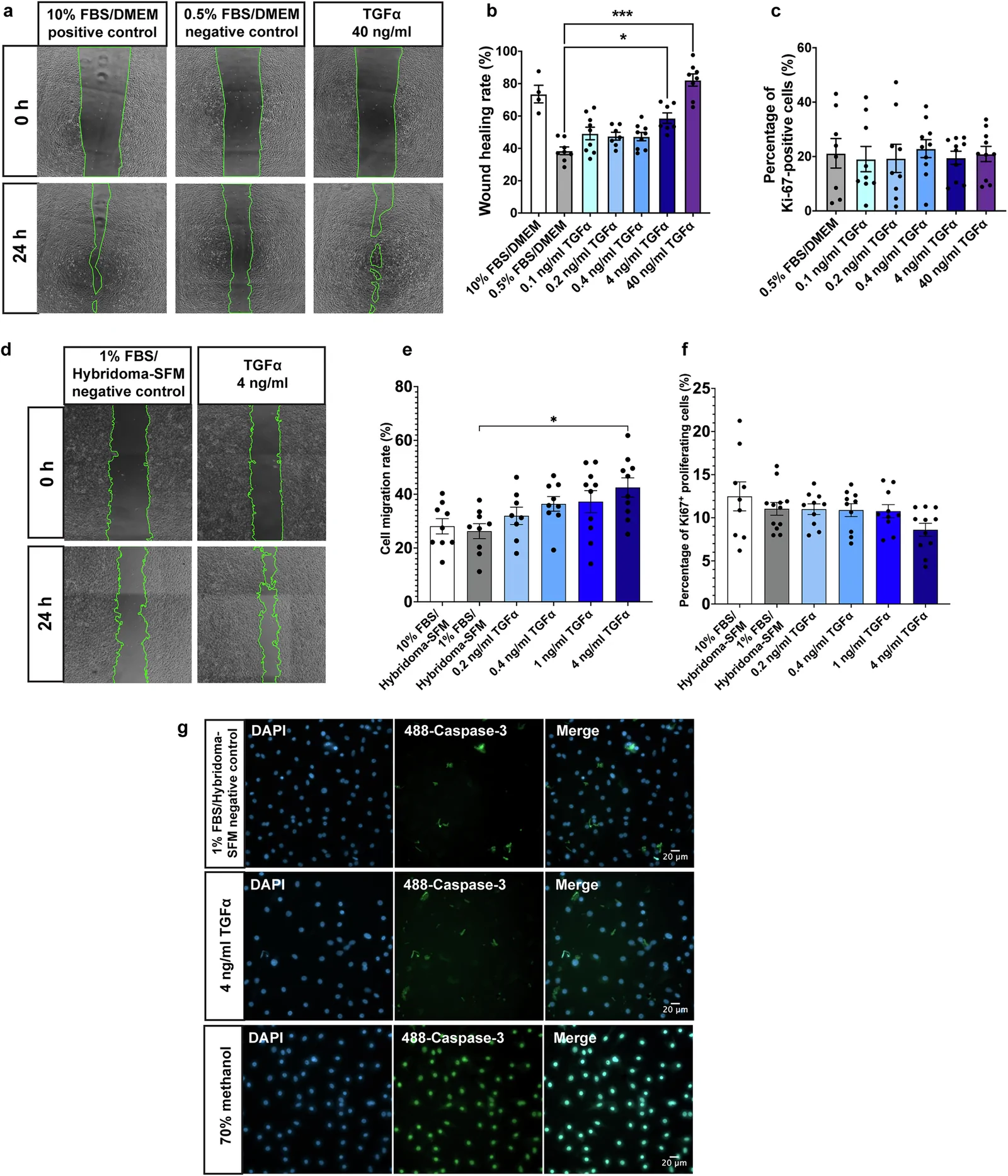
TGFα promotes wound healing and cell migration in A549 and primary human AT2 cells. **a** Representative images showing A549 cells treated with DMEM + 10% FBS (positive control), DMEM + 0.5% FBS (negative control), or 40 ng/ml TGFα at 0 and 24 hours post-scratch. Wound edges are marked with green lines. **b** Histogram of wound closure at 24 hours post-scratch in A549 cells treated with controls or increasing concentrations of TGFα (0.1, 0.2, 0.4, 4, and 40 ng/ml). **c** Percentage of Ki67-positive cells in A549 treated with varying TGFα concentrations, normalised to total DAPI-positive nuclei. *n* = 3 independent experiments; each experiment was run in duplicate or triplicate. Data are presented as mean ± SEM; Kruskal–Wallis with Dunn’s multiple comparisons test, *p < 0.05, ***p < 0.001. **d** Representative images of hAT2 cells treated with hybridoma medium + 1% FBS (negative control) or 4 ng/ml TGFα at 0 and 24 h post-migration. Green lines indicate cell-free areas. **e** Quantification of hAT2 migration at 24 h after treatment with hybridoma medium + 10% FBS (positive control), hybridoma medium + 1% FBS (negative control), or increasing TGFα concentrations (0.2, 0.4, 1, and 4 ng/ml). **f** Quantification of Ki67-positive hAT2 cells treated with different TGFα concentrations. *n* = hAT2 cells isolated from three donors; two or three technical replicates per experiment; each dot represents a technical replicate. Data were presented as mean ± SEM; Kruskal–Wallis with Dunn’s multiple comparisons test, *p < 0.05, ***p < 0.001. **g** Images showing hAT2 cells labelled with NucView® 488 Caspase-3 dye (green) to detect apoptotic cells at the experiment endpoint. Nuclei were counterstained with DAPI (blue). Hybridoma medium + 1% FBS served as a negative control; 70% methanol as positive control for apoptosis. Images were captured using an inverted Zeiss Axio Observer widefield epifluorescence microscope.

To evaluate the effects of TGFα, BMP2, and CXCL8 on wound healing and cell migration in alveolar epithelial cells, we treated A549 cells and primary human alveolar type 2 (hAT2) epithelial cells with recombinant proteins and performed scratch wound and cell migration assays, respectively. Treatment with TGFα significantly enhanced wound healing in A549 cells in a dose-dependent manner, with the maximal effect observed at 40 ng/ml (*p* < 0.0001) compared to the untreated control (DMEM supplemented with 0.5% FBS). Treatment with 4 ng/ml TGFα also significantly increased wound closure (*p* = 0.0149) (Fig. 5a, b). Additionally, immunostaining with the proliferation marker Ki67, followed by quantification of Ki67-positive cells, revealed no significant differences in cell proliferation across the various concentrations of TGFα (Fig. 5c). MTT assays further confirmed that TGFα did not affect cell viability at any concentrations tested. Cells treated with 70% methanol were used as a positive control for dead cells, which exhibited significant cell death compared to the untreated control (*p* = 0.0003) (Fig. S4).

Next, we replicated the scratch assay in human primary alveolar type 2 (hAT2) cells isolated from resected lung tissue, following a previously described method^30^. hAT2 cells exhibited low wound closure (10.5 to 20.8%) (Fig. S5), likely due to the removal of collagen-I matrix in the scratch area, which impaired hAT2 migratory capacity. We then employed a cell migration assay to further assess the effect of TGFα on hAT2 cells. Based on the dosage-response observed in the scratch assay (Fig. S5), we narrowed the range of TGFα concentrations for subsequent experiments to 0.2 to 4 ng/ml. Consistent with findings in A549 cells, TGFα treatment enhanced hAT2 migration in a concentration-dependent manner, with the most pronounced effect observed at 4 ng/ml TGFα (42.5% cell migration rate) compared to 26.3% in the control (*p* = 0.0314) (Fig. 5d, e). TGFα treatment also did not significantly impact cell proliferation (Fig. 5f), and labelling of hAT2 cells with NucView 488 caspase-3 dye indicated that TGFα did not induce apoptosis (Fig. 5g). MTT assays confirmed that cell viability was maintained in TGFα-treated cells. Cells treated with 70% methanol served as a positive control for dead cells, demonstrating a significant reduction in cell viability compared to the untreated control (*p* < 0.0001) (Fig. S6).

The effects of BMP2 and CXCL8 on wound healing in A549 cells were also investigated; however, no significant differences were observed between treated and untreated cells at any concentrations tested (Fig. S7a, b). MTT assays confirmed that the concentrations of BMP2 and CXCL8 used in these experiments did not affect cell viability (Fig. S7c, d).

### TGFα does not modulate cell migration or proliferation in human lung fibroblasts and microvascular endothelial cells

**Fig. 6:**
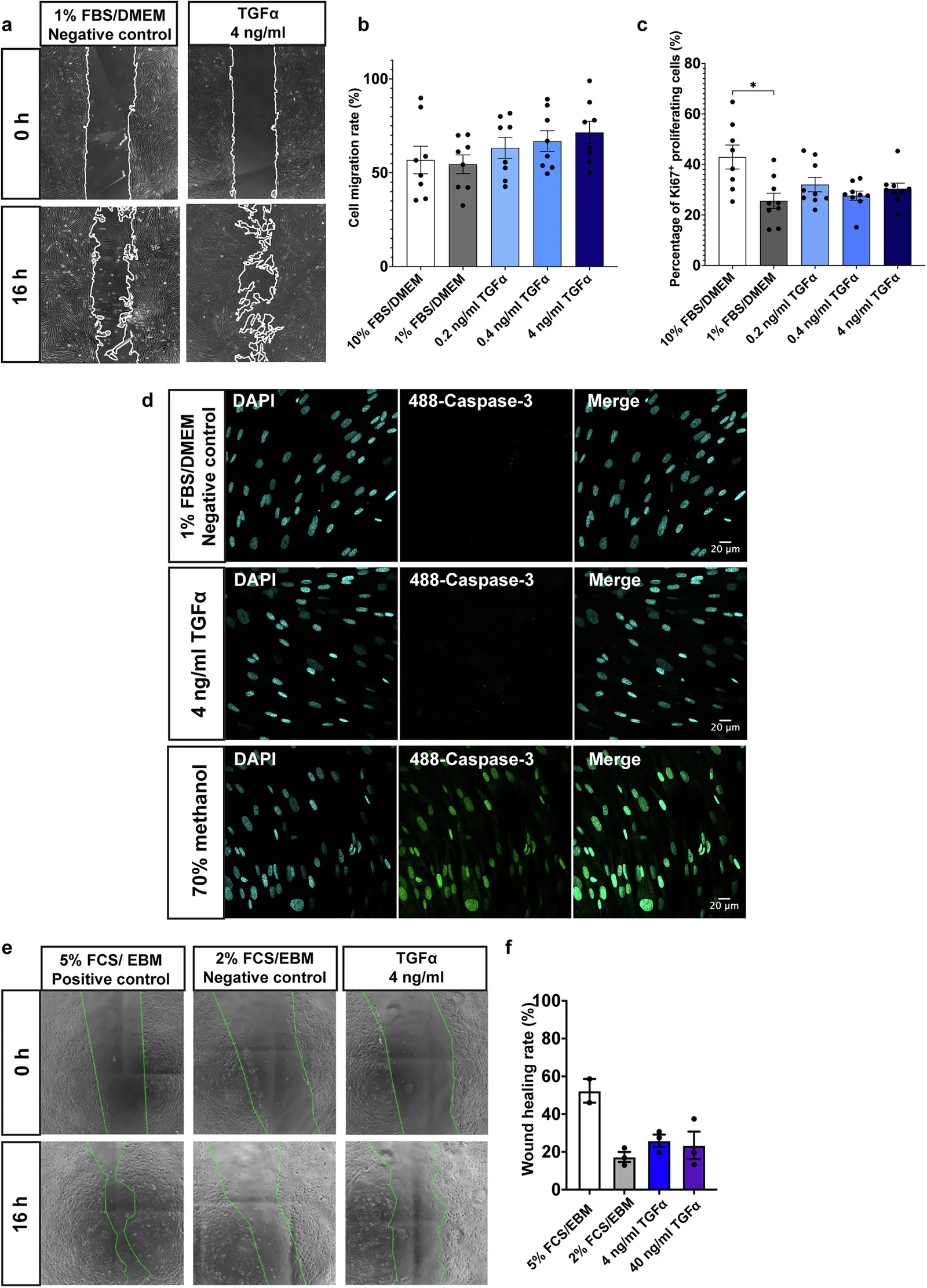
TGFα does not affect cell migration in HLFs or HPMECs. **a** Representative images of HLFs treated with DMEM + 1% FBS (negative control) or 4 ng/ml TGFα at 0 and 16 h post-scratch. White lines indicate wound areas. **b** Histogram illustrating cell migration rates at 16 h in HLFs incubated with DMEM + 10% FBS (positive control), DMEM + 1% FBS (negative control), and varying concentrations of recombinant TGFα (0.2, 0.4, and 4 ng/ml). **c** Percentage of Ki67-positive HLFs after treatment with different concentrations of TGFα. *n* = HLFs isolated from three donors; two or three technical replicates per experiment; each dot represents a technical replicate. Data were presented as mean ± SEM; Kruskal–Wallis with Dunn’s multiple comparisons test, *p < 0.05. **d** Representative images of HLFs stained with NucView® 488 Caspase-3 dye (green) and DAPI (cyan). DMEM + 1% FBS served as negative control; 70% methanol as positive control. Images were captured on a Leica SP8 inverted confocal microscope with a HC PL APO 40×/1.30 oil objective lens. **e**, **f** Wound closure at 0 and 16 h (**e**), and quantification of wound healing (**f**) in HPMECs treated with EBM + 5% FCS (positive control), EBM + 2% FCS (negative control), 4 or 40 ng/ml TGFα. Green lines indicate wound edges. *n* = 1 experiment; two or three technical replicates per experiment; each dot represents a technical replicate. Data were presented as mean ± SEM.

To determine whether TGFα also influences cell migration in other alveolar cell populations, cell migration and scratch wound assays were performed on primary human lung fibroblasts (HLFs) and HPMECs, respectively. Our results demonstrated that TGFα did not significantly affect cell migration in HLFs (Fig. 6a, b). Furthermore, immunostaining for the proliferation marker Ki67 revealed no effect of TGFα on cell proliferation. HLFs cultured in DMEM supplemented with 10% FBS exhibited a significantly higher percentage of Ki67-positive cells (42.9%) compared to control cells grown in DMEM containing reduced FBS (25.7%) (*p* = 0.0172) (Fig. 6c). As shown in Fig. 6d, no apoptotic cells were detected in either control or TGFα-treated HLFs, whereas cells treated with 70% methanol served as a positive control (Fig. 6d). Similarly, TGFα had no effect on wound healing in HPMECs (Fig. 6e, f). Cell viability assays confirmed that TGFα treatment did not influence metabolic activities in either HLFs or HPMECs (Fig. S8a, b).

In summary, our findings demonstrate that the pro-migratory and wound healing effects of TGFα are specific to alveolar epithelial cells.

### TGFα is associated with epithelial EGFR activation in response to endothelial angiogenesis

**Fig. 7:**
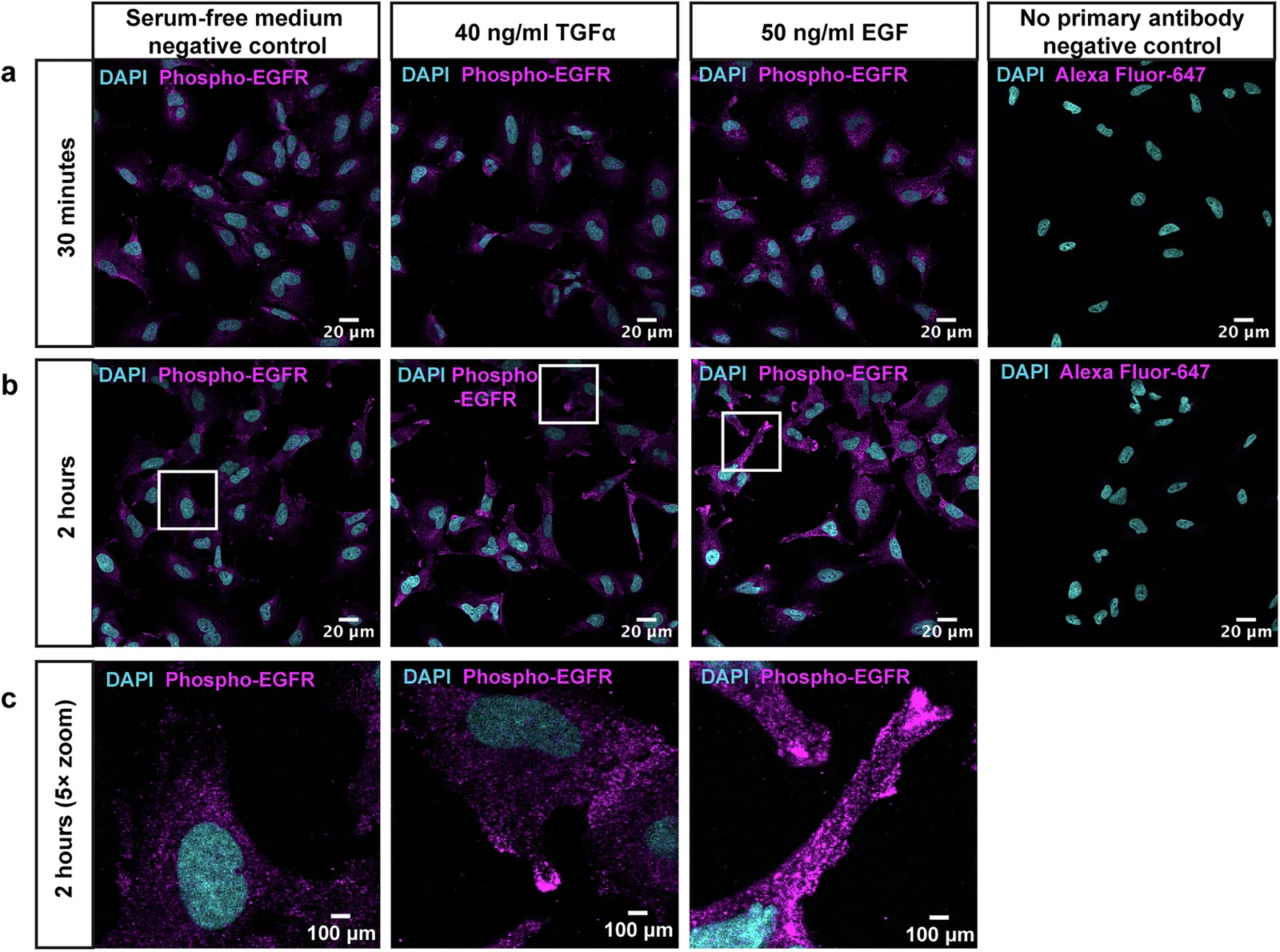
EGFR activation in A549 cells treated with exogenous TGFα or EGF. **a**–**c** Representative immunofluorescence images of untreated A549 cells (serum-free medium, negative control) or treated with 40 ng/ml TGFα or 50 ng/ml EGF for 30 min (**a**) or 2 h (**b**, **c**). Cells were immunostained for phospho-EGFR Y1068 (magenta), and nuclei were counterstained with DAPI (cyan). Images were captured using a Leica SP8 inverted confocal microscope with a HC PL APO 40×/1.30 oil objective lens. *n* = 2 independent experiments; three technical replicates per experiment.

Given that TGFα is a known ligand of EGFR, we next investigated whether TGFα-induced epithelial wound healing is associated with activation of this pathway. To establish conditions under which EGFR activation could be reliably detected, A549 cells were treated with recombinant TGFα or epidermal growth factor (EGF; another EGFR ligand) and assessed by immunostaining for phospho-EGFR. No clear changes in phospho-EGFR staining were observed at 30 min (Fig. 7a). However, after 2 h of stimulation, both TGFα- and EGF-treated cells exhibited increased membrane-associated phospho-EGFR compared with serum-free controls (Fig. 7b, c), indicating that EGFR activation is detectable at this time point.

We next sought to detect TGFα secretion from HPMECs. ELISA analysis of conditioned media collected from EtOH- or RA-treated HPMECs at 4 and 16 h post-treatment failed to detect TGFα. Notably, although the TGFα standard provided with the ELISA kit produced a valid standard curve, commercially available recombinant TGFα spiked into culture media was also not detected, suggesting a technical limitation of the assay under these conditions (Table S5).

**Fig. 8:**
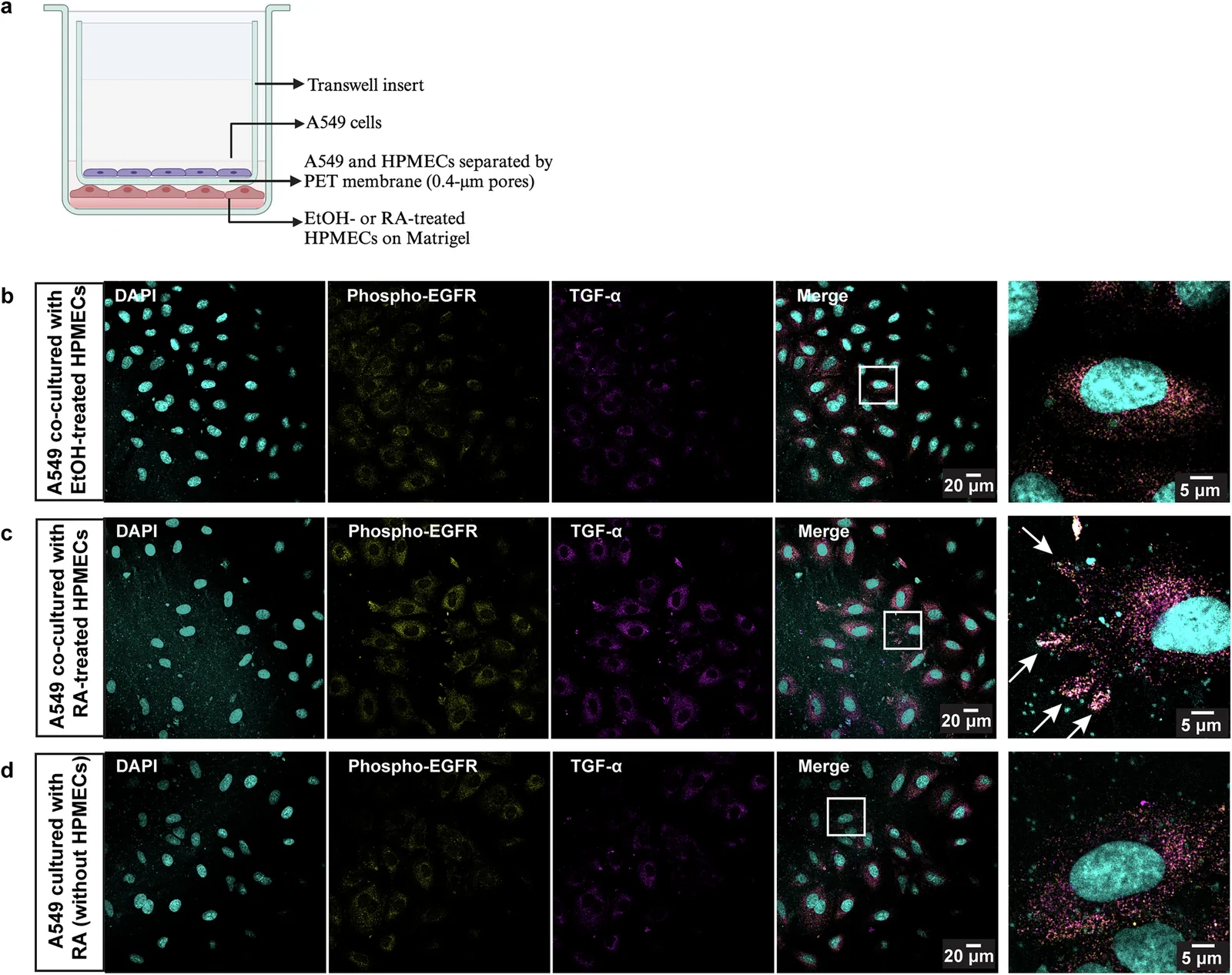
Membrane-associated TGFα and EGFR activation in A549 cells co-cultured with RA-treated HPMECs. **a** Schematic of the HPMEC–A549 transwell co-culture system. A549 cells were cultured in the upper chamber and co-cultured with HPMECs seeded on Matrigel in the lower chamber with either 0.1% EtOH or 10 μM RA. The diagram was created in BioRender. **b**, **c** Representative immunofluorescence images of A549 cells following 2 h of co-culture with EtOH control-treated HPMECs (**b**), RA-treated HPMECs (**c**). Higher magnification images are shown in the right panel. **c** Arrows indicate the distribution of TGFα and phospho-EGFR at the cell membrane in A549 cells co-cultured with RA-treated HPMECs. **d** Control experiment showing A549 cells treated with RA in the absence of HPMECs. A549 cells were immunostained for phospho-EGFR (yellow) and TGFα (magenta). Nuclei were counterstained with DAPI (cyan). Images were captured on a Leica SP8 inverted confocal microscope with a HC PL APO 40×/1.30 oil objective lens. *n* = 2 independent experiments; three technical replicates per experiment.

Given this limitation, we adopted an alternative approach to assess TGFα activity and distribution in an A549-HPMEC transwell co-culture system. A549 cells were co-cultured with RA- or EtOH-treated HPMECs on Matrigel, followed by immunostaining for phospho-EGFR and TGFα (Fig. 8a). Increased membrane-associated phospho-EGFR was observed in A549 cells co-cultured with RA-treated HPMECs, accompanied by enrichment of TGFα signal that appeared to co-localise with phospho-EGFR at the cell membrane. This pattern was not observed in A549 cells co-cultured with EtOH-treated HPMECs (Fig. 8b, c). Importantly, direct treatment of A549 cells with RA in the absence of HPMECs did not reproduce this distribution of phospho-EGFR or TGFα, supporting a paracrine mechanism (Fig. 8d).

## Discussion

Here, we conducted a microarray to identify candidate signalling factors upregulated in RA-treated HPMECs undergoing angiogenesis. Among these, CXCL8, TGFα and BMP2 were selected for further investigation based on their degree of upregulation and biological relevance. TGFα specifically induced repair and migration of both A549 and human primary AT2 cells, whereas CXCL2 and BMP2 did not. In contrast, TGFα had no effect on the repair or proliferation of either primary HLFs or HPMECs. Subsequently, using a co-culture approach, we found that RA-induced HPMEC angiogenesis was associated with activation of EGFR signalling and increased membrane-associated TGFα in adjacent A549 cells, supporting a paracrine interaction between endothelial and epithelial cells.

It has long been appreciated that RA is a powerful regenerative agent across organs and species^31,32^. Interestingly, a recent study in the skin identified a pivotal role of RA in regulating stem cell fate during wound repair by modulating lineage plasticity; a transient state that bestows tissue stem cells with fate flexibility and is important in the repair response to injury^33^. In the lung, RA is critical for development and has been implicated in alveolar regeneration^11,14^. Epidemiological studies further demonstrate that vitamin A deficiency has detrimental effects not only on the lungs but also on other organ systems^34^. Promising data from animal experiments and population studies led to clinical trials being conducted with the aim of determining whether RA might be an effective treatment for patients with emphysema. Although the results of these first human lung regenerative clinical trials were largely disappointing and failed to meet their primary end points, in one trial, involving patients with CT-confirmed smoking-related emphysema, post-hoc subgroup analysis found that patients with predominantly lower-zone disease experienced reduced declines in FEV1, gas diffusion, and exercise capacity compared with placebo^35^. These findings highlight the need for a deeper mechanistic understanding of RA-driven repair processes.

We previously showed that instead of directly promoting epithelial repair, RA induced angiogenesis in HPMECs^15^. We therefore hypothesised that RA may indirectly influence alveolar epithelial repair through paracrine signalling associated with endothelial angiogenesis. To investigate this, we combined the use of human cell-based and 3D ex vivo models with detailed mechanistic studies of RA-driven responses in the lung. Our findings support this hypothesis by demonstrating that RA-induced angiogenesis in HPMECs is associated with upregulation of TGFα and activation of EGFR signalling in alveolar epithelial cells. Furthermore, exogenous TGFα enhanced epithelial repair responses, suggesting a potential role for TGFα in endothelial–epithelial crosstalk.

TGFα is one of seven ligands that bind to EGFR with varying affinities^36^. Considerable evidence has demonstrated a role for EGF ligands and EGFR signalling in lung repair^37^. However, although TGFα is expressed in the lungs throughout the life course, its role in repair and regeneration remains unclear. While overexpression studies in mice have linked TGFα to pathological remodelling, including alveolar airspace enlargement and fibrosis, these effects appear to be dose-dependent^17^. TGFα is also upregulated in lung diseases characterised by tissue damage, including idiopathic pulmonary fibrosis (IPF), COPD, and in post-COVID patients (39–41). Although this has often been interpreted as detrimental, it could equally reflect a context-dependent adaptive repair response, with TGFα contributing positively to lung regeneration. Recently, Li and colleagues showed that lipopolysaccharide (LPS) injury induces upregulation of TGFα/EGFR signalling, leading to abnormal elastin deposition and alveolar simplification; inhibition of EGFR with erlotinib mitigated these effects on alveoli, underscoring the need for tight regulation of TGFα levels and highlighting the TGFα-EGFR axis as a potential therapeutic target to modulate alveolar regeneration^17,38^.

Consistent with a context-dependent role, in our current study, *TGFA* mRNA was not increased in RA-treated HPMEC monolayers but was upregulated during RA-induced angiogenesis. This suggests that *TGFA* induction is not a direct effect of RA treatment but rather a response linked to the angiogenic state, supporting our hypothesis that endothelial activation during angiogenesis promotes paracrine signalling that mediates epithelial repair. This contrasts with a previous study reporting suppression of *TGFA* expression in upper airway epithelial cells and airway explants treated with retinol^39^. Notably, our study used retinoic acid (a derivative of retinol) at 10 μM compared with 0.1 μM retinol in Miller’s work and focused on alveolar cells rather than airway epithelial cells or tissues, suggesting that *TGFA* transcriptional responses to retinoids are tightly regulated, both dose- and context-dependently.

The validity of the microarray data was confirmed by the enrichment of RA signalling pathways in HPMECs 4 h after RA treatment. GO analysis highlighted two major aspects of RA-induced changes: first, reorganisation of actin filaments, which governs cell adhesion and migration, processes essential for wound healing and tissue repair, and second, changes in epithelial cell behaviour, supporting the idea of crosstalk between HPMECs and epithelial cells in response to exogenous RA exposure.

However, in the current study, we were unable to detect TGFα in supernatants collected from A549-HPMEC co-cultures. While the ELISA assay produced a valid standard curve, TGFα was not detected in co-culture supernatants, or in culture media supplemented with recombinant TGFα, which was included as a positive control (Table S5). This suggests methodological limitations, potentially suboptimal timing of supernatant collection or limited recognition of specific TGFα isoform(s) by the antibodies used. Notably, multiple isoforms of TGFα are annotated in Ensembl, which could contribute to antigen-antibody mismatch. Despite this limitation, we observed membrane-associated TGFα together with activation of EGFR signalling in A549 cells exposed to RA-treated HPMECs undergoing angiogenesis in co-culture, supporting a paracrine mechanism.

Ideally, mechanistic confirmation could be obtained by blocking a TGFα-specific EGFR phosphorylation site. However, no known TGFα-specific EGFR phosphorylation sites have been identified to date^40^. Interrogating the specific contribution of an individual EGFR ligand, such as TGFα, is further complicated by the well-established redundancy within the EGFR ligand family. EGFR signalling is mediated by a network of ligands with overlapping and partially redundant functions in epithelial systems^41,42^. This is supported by genetic studies demonstrating that combined loss of multiple EGFR ligands does not phenocopy EGFR deficiency. Specifically, mice lacking EGF, TGFα and amphiregulin remain viable and display relatively mild developmental defects, in contrast to EGFR-null mice, which exhibit severe epithelial abnormalities and perinatal lethality. Notably, despite the absence of these three ligands, ERK activation in mammary epithelial structures was largely preserved, indicating that EGFR signalling can be maintained through compensatory activity from remaining ligands^43^. Consistent with this, experimental studies have demonstrated that disruption of individual EGFR ligands is insufficient to abrogate downstream signalling, whereas simultaneous ablation of multiple ligands is required to significantly impair pathway activation, highlighting the inherent functional redundancy of the EGFR ligand network^40^.

In line with this, we attempted to block TGFα using a neutralising antibody in our system. However, this did not reduce EGFR activation (Fig. S9), consistent with compensatory activity from other EGFR ligands. While global EGFR inhibition (e.g., using tyrosine kinase inhibitors such as erlotinib) has been shown to modulate epithelial repair, this approach would not allow us to address the specific role of TGFα. Instead, it would introduce significant confounding effects by simultaneously blocking all EGFR ligands, thereby broadly impairing epithelial repair processes, given the well-established role of EGFR signalling in epithelial cell migration, proliferation, and wound healing.

To further assess the functional relevance of endothelial-derived signals, we evaluated epithelial wound closure in response to conditioned media from RA-treated HPMECs. However, under these experimental conditions, no significant differences in wound healing rates were observed (Fig. S10). Notably, our dose–response experiments demonstrated that TGFα promotes epithelial migration within a defined concentration range, suggesting that a threshold level of stimulation is required to elicit measurable effects on wound closure. In contrast, endothelial-derived TGFα in the co-culture system is likely present at lower abundance and may be spatially restricted or transient in nature. These conditions may be sufficient to activate EGFR signalling, as evidenced by increased membrane-associated phospho-EGFR, but insufficient to drive detectable changes in the functional assay. Consistent with this, membrane-bound pro-TGFα has been shown to activate EGFR on adjacent cells without requiring accumulation of soluble ligand^44^, and EGFR ligands are known to signal through localised juxtacrine/paracrine mechanisms^36^. These findings highlight the distinction between pathway activation and downstream functional outcomes and suggest that additional factors or more physiologically relevant systems may be required to fully recapitulate endothelial–epithelial repair dynamics.

In summary, this study shows that RA induces angiogenesis in lung endothelial cells, leading to activation of EGFR signalling in alveolar epithelial cells via paracrine mediators, including TGFα. More broadly, our findings highlight the importance of using physiologically relevant human models to uncover the detailed mechanisms of tissue repair required for the development of effective pharmacological regenerative medicine treatments.

## Methods

### Sex as a biological variable

Human tissue, for cell isolation, was obtained from both males and females.

### Mice

All animal maintenance and procedures were conducted in compliance with the requirements of the Animal (Scientific Procedures) Act 1986. Animal work was approved by the South Kensington AWERB committee at Imperial College London. Mice were housed in specific pathogen-free conditions and given food and water ad libitum. Wild-type female mice aged 10–12 weeks were purchased from Charles River (United Kingdom).

### Mouse precision-cut lung slices (PCLS) and acid injury

Mouse PCLS were generated following previously established protocols^45,46^. Briefly, adult mice were euthanised via intraperitoneal injection of pentobarbital. The anterior chest and neck wall were carefully dissected, and a small incision was made in the trachea below the cricoid cartilage. A 21 G rigid metal cannula was inserted into the trachea incision, and the lungs were inflated by injecting 1.2 ml of 2% (w/v) low-gelling agarose (Sigma-Aldrich, A9414) dissolved in 1× Hank’s Balanced Salt Solution (HBSS; Life Technology, 14025100) containing 1% HEPES buffer (Life Technology, 15630080). The chest cavity was cooled with ice to solidify the agarose.

The lungs and heart were excised and submerged in ice-cold HBSS/HEPES buffer. Lung lobes were carefully separated and sliced transversely at a thickness of 300 µm using an automated vibratome (Compresstome VF-300-0Z; Precisionary Instruments LLC) in ice-cold HBSS/HEPES buffer. PCLS were then transferred to 24-well plates containing ice-cold serum-free (SF) Dulbecco’s Modified Eagle Medium (DMEM)-GlutaMAX (Life Technologies, 31966021) with 1% penicillin-streptomycin (Merck, P0781). PCLS were incubated overnight at 37 °C in a 5% CO_2_ atmosphere and washed three times with warm SF-DMEM to remove any remaining agarose.

For acid injury, PCLS were treated with 0.1 M HCl (Fisher Chemical, 10053023) for 1 min at room temperature (RT). The HCl solution was then discarded, and PCLS were washed three times with phosphate-buffered saline (PBS; Sigma; D8537) to remove residual acid. PCLS were cultured in fresh SF-DMEM for 48 h before RNA extraction.

### Cell culture

Primary human pulmonary microvascular endothelial cells (HPMECs; Promocell, C12281) and human umbilical vein endothelial cells (HUVECs; Promocell, C12200) were cultured in Endothelial Cell Growth Medium MV (EGM-MV; Promocell, C22020) and Endothelial Growth Media (EGM; Promocell, C-22010), respectively. Cells were maintained at 37 °C in a 5% CO_2_ atmosphere. Cells were used between passages 3 and 7, showing no changes in morphology or baseline tube-forming capacity. HPMECs used in this study were sourced from two Caucasian female donors, aged 64 and 98. A549 cells, a human alveolar adenocarcinoma epithelial cell line, were purchased from the American Type Culture Collection (ATCC). Cells were cultured in DMEM-GlutaMAX (Gibco) supplemented with 10% foetal bovine serum (FBS; Sigma, F9665) and 1% penicillin/streptomycin at 37 °C with 5% CO_2_.

The research carried out in this manuscript adheres to the World Medical Association Declaration of Helsinki.

Primary human alveolar type 2 (hAT2) epithelial cells and human lung fibroblasts (HLFs) were isolated from histologically normal lung tissues obtained from patients undergoing surgical resection for suspected lung tumours. Lung tissue samples were collected from patients undergoing lung resection at the Royal Brompton Hospital (RBH), by Biobank sample co-ordinators within the RBH Clinical Research Facility. All samples were obtained following informed consent from patients. The research was conducted under the National Research Ethics Service (NRES) reference number: 20/SC/0142. The isolation protocol was based on a previously described method^30^ with slight modifications.

Briefly, lung tissue was perfused with HBSS (Invitrogen, 14025-050) to remove blood and debris, followed by digestion with 0.25% trypsin (Sigma, T8003) at 37 °C for 45 min. Trypsinisation was halted by adding FBS, and the tissue was finely chopped. DNase I (Sigma, DN25) was added to facilitate degradation of extracellular DNA to improve tissue dissociation. The resulting tissue suspension was passed through a tea strainer and a 40-μm mesh filter (Marathon Lab Supplies, 352340) to remove undigested tissue. The filtrate was centrifuged at 1300 rpm for 10 min at 12 °C. The pellet was resuspended in hybridoma serum-free medium (SFM; Gibco, 11570416) containing 50 μg/ml DNase I and incubated at 37 °C for 45 min to remove immune cells.

After incubation, the supernatant was collected and centrifuged again at 1300 rpm for 10 min at 12 °C. The pellet was resuspended in hybridoma medium containing 10% FBS and incubated at 37 °C for 2 h. Cells adhering to the dishes after this period were identified as HLFs, which were subsequently cultured in DMEM-GlutaMAX supplemented with 10% FBS for downstream experiments. The supernatant containing non-adherent cells was centrifuged at 1300 rpm for 10 min at 12 °C, and the resulting pellet, containing hAT2 cells, was resuspended and cultured in hybridoma medium with 10% FBS. hAT2 cells were not passaged, and all experiments involving these cells were completed within 68 hours of seeding. HLFs were used between passages 3–7 for experiments.

The specificity of isolated hAT2 cell populations was confirmed by immunostaining with alveolar cell type-specific markers. The hAT2 cells were positive for pan-cytokeratin (a pan-epithelial marker) and Pro-SPC (an AT2 cell marker), but negative for vimentin (a fibroblast marker) and podoplanin (PDPN; an AT1 cell marker) (Fig. S11).

### Pharmacological treatment

Retinoic acid (RA; Sigma, R2625) used in this study was dissolved in absolute ethanol (EtOH; Sigma-Aldrich, 51976). Recombinant human TGFα (catalogue number: 239-A1-100), BMP2 (catalogue number: 355-BM-010), and CXCL8 (catalogue number: 208-IL-010) were obtained from R&D Systems and reconstituted according to the manufacturer’s instructions. Human EGF was sourced from Promocell (catalogue number: C-39220).

### Matrigel-based angiogenesis assay and tube quantification

Angiogenesis μ-slides (Ibidi, 81506) or 24-well plates (Ibidi, 2401) were precoated with growth factor-reduced Matrigel (BD Biosciences, 354230), diluted to a concentration of 5 mg/ml with prechilled endothelial cell basal medium (EBM; Promocell, C-22220) without supplements. The plates were incubated at 37 °C for 1 h to allow the Matrigel to solidify. HPMECs were serum-starved for 24 h prior to conducting the tube formation assays. Cells were seeded at a density of 4750 cells per well for the angiogenesis μ-slides and 70,000 cells per well for the 24-well plates. Cells were treated with either the vehicle (EtOH) or 10 μM RA.

For time-lapse imaging of tube formation, the μ-slide was immediately transferred to a preequilibrated, humidified incubator chamber (37 °C, 5% CO_2,_ and ~21% oxygen levels) of an inverted Zeiss Axio Observer widefield epifluorescence microscope. Imaging was performed over a 4-h period using an EC Plan-Neofluar 10×/0.30 objective lens. The total tube length and the total number of nodes per field of view were quantified using FIJI software. Data were expressed as the mean total tube length or total number of nodes per field of view.

### RNA extraction and quantitative real-time PCR (qRT-PCR)

Mouse PCLS were homogenised using a FastPrep-24TM Tissue Homogeniser (MP Biomedicals), and total RNA was extracted with the RNeasy mini kit (Qiagen, 74104) following the manufacturer’s protocols. For each experimental condition, RNA samples were pooled from three PCLS, and a total of three RNA samples were prepared per condition. RNA was also extracted from HPMEC monolayers using the same RNeasy mini kit. For HPMECs cultured on Matrigel, Matrigel was first dissolved using Cell Recovery Solution (Corning, 354253) according to the manufacturer’s instructions, prior to RNA extraction. RNA concentration and integrity were assessed using the TapeStation 2200 system (Agilent), and only samples with an RNA integrity number (RIN) greater than 8 were used for downstream cDNA synthesis and qRT-PCR.

Approximately 200 ng of total RNA was reverse transcribed into cDNA using the High-Capacity cDNA Reverse Transcription kit (Applied Biosystems) according to the manufacturer’s instructions. Quantitative RT-PCR was then performed using TaqMan Fast Advanced Master Mix (Life Technologies) on a 7500 Fast Real-Time PCR system (Applied Biosystems). The housekeeping gene *B2m* was used as a reference for normalisation. Relative gene expression levels were calculated using the 2^−ΔΔCT^ method. Each sample was tested in triplicate, with four biological replicates derived from four different mice.

The following TaqMan primers (Life Technologies) were used in this study: mouse *B2m* (Mm00437762_m1), human *B2M* (Hs00984230_m1), *Rara* (Mm01296312_m1), *Rarb* (Mm01319677_m1), *Rarg* (Mm00441083_m1)*, Aldh1a1* (Mm00657317_m1), *Aldh1a2* (Mm00501306_m1), *Aldh1a3* (Mm00474049_m1), *Cyp26a1* (Mm00514486_m1), *Cyp26b1* (Mm00558507_m1), *Cyp26c1* (Mm03412454_m1), *TGFA* (Hs00608187_m1)*, CXCL8* (Hs00174103_m1)*, BMP2* (Hs00154192_m1)*, HOXA3* (Hs00601076_m1), and *HOXA5* (Hs00430330_m1).

### Microarrays and raw data processing

Microarray service was provided by UK Bioinformatics Ltd. (UKB, UK). Total RNA was extracted from HPMECs cultured on Matrigel using Trizol reagent (Invitrogen, UK). RNA quality was assessed using the Agilent 2100 Bioanalyzer. For each treatment and time point, four RNA samples were prepared from independent experiments, with each sample pooled from three technical replicates (see Table S6 for sample details).

cDNA was synthesised using the Ovation^®^ Pico WTA System V2 and labelled with the Encore Biotin Module, following the manufacturer’s protocols (Nugen, USA). The labelled cDNA was hybridised to Affymetrix Human U133 Plus 2.0 Gene Chips (Affymetrix) according to the manufacturer’s instructions. The resulting microarray data were generated in an Affymetrix CEL format and processed using the Transcriptome Analysis Console (TAC) software v4.0.2 (Thermo Scientific).

Raw data normalisation and summarisation were performed using the MAS5 algorithm within the TAC software. Gene expression analysis was conducted using the human genome reference assembly hg19. Differential expression was defined by a fold change threshold of >1.5 or <−1.5, and a false discovery rate (FDR)-adjusted *p* value of <0.05. Statistical significance was calculated using moderated t-statistics from the empirical Bayes (ebayes) method within an ANOVA framework, allowing for robust identification of differentially expressed genes between RA-treated and EtOH-treated control samples. FDR correction was applied using the Benjamini–Hochberg algorithm to control for multiple testing.

### Gene set enrichment analysis (GSEA)

GSEA was performed to identify pathways and biological processes significantly associated with upregulated genes. Pathway enrichment analysis was conducted using PANTHER version 19.0 (www.pantherdb.org/), focusing on PANTHER Pathways and Reactome Pathways. For the enrichment of Gene Ontology (GO) biological processes, Cytoscape software version 3.10.2 was utilised, in conjunction with the STRING enrichment tool. Enriched pathways and biological processes were ranked according to their statistical significance.

### Scratch wound and cell migration assays

For the scratch wound assay, 45,000 HUVECs, HPMECs, or A549 cells were seeded onto eight-well Permanox chamber slides (Fisher Scientific; 16250681), precoated with 2 μg/ml fibronectin (Sigma, F1141) for HUVECs and HPMECs, or 50 μg/ml rat-tail collagen I (Gibco, A1048301) for A549 cells. Cells were cultured in their respective complete media—EGM for HUVECs, EGM-MV for HPMECs, and DMEM-GlutaMAX supplemented with 10% FBS for A549, and incubated overnight at 37 °C with 5% CO_2 to_ reach confluence_._ A scratch wound was made across the centre of the confluent monolayer using a P1000 pipette tip. Wells were gently rinsed with PBS to remove dislodged cells, followed by the addition of appropriate treatments.

For RA treatments, HUVECs were exposed to 3, 5, 10, 20, or 50 μM RA, diluted in EBM. A549 cells were treated with 10 μM of RA in SF-DMEM. Since RA was reconstituted in EtOH, control media containing 0.1% EtOH in either EBM or SF-DMEM were used for HUVECs and A549 cells, respectively. Positive control media for enhanced wound healing consisted of EGM with 3% FCS for HUVECs and DMEM supplemented with 10% FBS for A549 cells.

For the cell migration assay, hAT2 and HLFs were plated using two-well silicone culture inserts (Ibidi, 80209) adhered to 50 μg/ml rat-tail collagen I-coated 13 mm coverslips placed in 24-well plates. Freshly isolated hAT2 cells were seeded at a density of 250,000 cells per well, while HLFs were plated at 15,000 cells per well. For HLFs, the silicone inserts were removed after overnight incubation. For hAT2 cells, debris and dead cells were gently washed off with PBS 24 h post-seeding, and fresh hybridoma SFM supplemented with 10% FBS was added, followed by a further 24-h incubation before insert removal. TGFα treatments were administered at appropriate concentrations in DMEM with 1% FBS for HLFs or in hybridoma SFM with 1% FBS for hAT2 cells.

Cells in both assays were imaged at time points *t* = 0 h and the endpoint at *t* = 19 or 24 h using an inverted Zeiss Axio Observer widefield epifluorescence microscope housed in a preequilibrated, humidified incubator chamber (37 °C, 5% CO_2_, ~21% oxygen). Images were captured using an EC Plan-Neofluar 10×/0.30 objective lens, and tiling was employed to image the cell-covered areas. Wound healing percentage was quantified using a FIJI macro developed by Steve Rothery (Facility for Imaging by Light Microscopy, Imperial College London) or through manual analysis. For manual quantification, the wound areas were outlined with the freehand tool in FIJI, and the percentage healed was calculated by subtracting the endpoint area from the area at *t* = 0 h. Three independent experiments were conducted, and each experiment was run in triplicate.

### Immunofluorescence and imaging

Cells were fixed in 4% paraformaldehyde (PFA) for 10 min at RT, followed by PBS. Permeabilisation was carried out using 0.2% (v/v) Triton X-100 in PBS at RT for 5 min. Blocking was then performed for 1 h at RT using PBSBT buffer, which contains 3% bovine serum albumin (BSA) and 0.1% Triton X-100 in PBS. After blocking, cells were incubated overnight at 4 °C with primary antibodies diluted in PBSBT blocking buffer. The following primary antibodies were used in this study: rabbit anti-vimentin (Bioorbyt, orb304659; 1:200), mouse anti-pan-cytokeratin (Sigma-Aldrich, C2931; 1:200), rabbit anti-Pro-SPC (Abcam, ab90716; 1:300), hamster anti-PDPN (Developmental Studies Hybridoma Bank, 8.1.1; 1:50), rat anti-Ki67 (eBioscience, 14-5698-82; 1:500), rabbit anti-phospho-EGFR Y1068 (Cell Signalling Technology, 3777S; 1:100), and goat anti-TGF-alpha (R&D Systems, AF-239-NA; 1:30).

Following three washes with PBS, cells were incubated for 1 h at RT with rhodamine phalloidin (Biotium, 00027; 1:300) and species-specific Alexa Fluor 488, 594, and 647 secondary antibodies (Thermo Fisher Scientific; 1:500). After further PBS washes, cell nuclei were stained with DAPI (Thermo Scientific, 62249). Coverslips were mounted with ProLong Gold Antifade Mountant (Invitrogen, P36930). Imaging was performed using a Leica SP8 inverted confocal microscope equipped with a HC PL APO 10×/0.40 air objective lens or a HC PL APO 40×/1.30 oil objective lens. For some experiments, channel colours were changed during image post-processing to improve visual clarity.

### MTT assay

To evaluate the impact of pharmacological treatments on cell viability, an MTT assay was conducted according to the manufacturer’s instructions (Roche, 11465007001). Briefly, 150 μl of 10% MTT solution diluted in serum-free media was added to each well of a 96-well plate and incubated at 37 °C for 45 min. The resulting formazan crystals, which formed in viable cells, were solubilised by adding an equal volume of DMSO and incubating at 37 °C for 10 min. About 100 μl of eluted formazan solution was measured spectrophotometrically, with absorbance recorded at 570 nm and corrected at 690 nm using a plate reader.

### Caspase-3 assay

To identify apoptotic cells, the NucView® 488 Caspase-3 assay kit (Biotium; 30029-T) was employed, following the manufacturer’s protocols. At the end point of pharmacological treatments, cells were incubated with 5 μM NucView 488 substrate (prepared as a 1:40 stock dilution) in the dark at RT for 30 min. After incubation, the dye was removed, and the cells were gently washed three times with PBS. Cells were then fixed with 4% PFA for 10 minutes at RT. After fixation, the PFA was discarded, and the cells were washed three times with PBS. Cells were counterstained with DAPI at RT for 5 min and washed once with PBS. Cells were then mounted using ProLong® Gold Antifade Reagent and imaged on the same day using a Leica SP8 inverted confocal microscope equipped with a HC PL APO 40×/1.30 oil objective lens.

### Transwell insert-based coculturing of HPMECs and A549

HPMECs and A549 cells were co-cultured as illustrated in Fig. 8a. A549 cells were plated at a density of 2 × 10⁴ cells per well onto 24-well transwell inserts with 0.4-μm pore polyethylene terephthalate (PET) membrane (Brand, BR782711), precoated with 50 µg/ml rat-tail collagen-I. Cells were maintained in DMEM supplemented with 10% FBS and incubated overnight at 37 °C in a humidified atmosphere containing 5% CO_2_.

The following day, HPMECs were seeded onto Matrigel in the lower chamber and cultured in EBM as described in the section “Matrigel-based angiogenesis assay and tube quantification”. HPMECs were treated with either 0.1% EtOH (vehicle control) or 10 μM RA.

Prior to co-culture, the medium in the transwell inserts containing A549 cells was removed, and cells were washed three times with PBS to remove any residual serum. Inserts were then replenished with EBM supplemented with 0.2% FCS. After 4 h of RA or EtOH treatment, transwell inserts containing A549 cells were placed into wells containing HPMECs, initiating co-culture for 2, 4, or 16 h.

After the 2-h timepoint, A549 cells in the upper chamber were fixed and subjected to immunostaining for phospho-EGFR and TGFα. Conditioned media from the lower chamber were collected at 4and 16 h for quantification of extracellular TGFα using the Human TGF-alpha DuoSet ELISA (R&D Systems, DY239), according to the manufacturer’s instructions.

### Statistical analysis

All graphs and statistical tests were produced using GraphPad Prism 10. Data were expressed as mean ± standard error of mean (SEM). The D’Agostino–Pearson test was employed to assess the normality of the data. For non-parametric datasets, the Mann–Whitney *U*-test was utilised for comparisons between two groups, while comparisons involving more than two groups were performed using the Kruskal–Wallis test followed by Dunn’s multiple comparison test. The specific statistical tests applied, sample size (*n*), and the number of experiments conducted are detailed in the figure legends. A *p* value of less than 0.05 was considered statistically significant.

### Ethics approval

Lung tissue samples were collected from male and female patients undergoing lung resection at the Royal Brompton & Harefield hospital (RBHT), by Biobank sample co-ordinators within the RBHT Clinical Research Facility (CRF). All samples were obtained following informed consent from patients. The research was conducted using the RBHT Biobank ethics number: 20/SC/0142.

## Supplementary information

Supplementary Information

Supplementary Data 1

Video S1

Video S2

## Data Availability

The microarray data generated or analysed during the current study are available in the Gene Expression Omnibus (GEO) repository under the accession number GSE307491.
