# Supplementary material for: TGF-alpha/EGFR signalling mediates retinoic acid-induced lung repair
Source: NPJ Regen Med. 2026 Jul 4;11:46. doi: 10.1038/s41536-026-00492-9 (PMC13710761; doi:10.1038/s41536-026-00492-9)
Supplement: Supplementary file 1 [file 41536_2026_492_MOESM1_ESM.pdf]

Video S1. Time-lapse video of HPMECs treated with 10 µM of RA undergoing angiogenesis. Imaging was performed in a pre-equilibrated, humidified incubator chamber maintained at 37°C with 5% CO_2 and approximately 21% oxygen. Images were captured every 20 minutes over a period of 4 hours using an inverted Zeiss Axio Observer widefield epifluorescence microscope with an EC Plan-Neofluar 10×/0.30 objective lens. Snapshot images show HPMECs at 40 minutes (left panel) and 4 hours (right panel) after RA treatment.

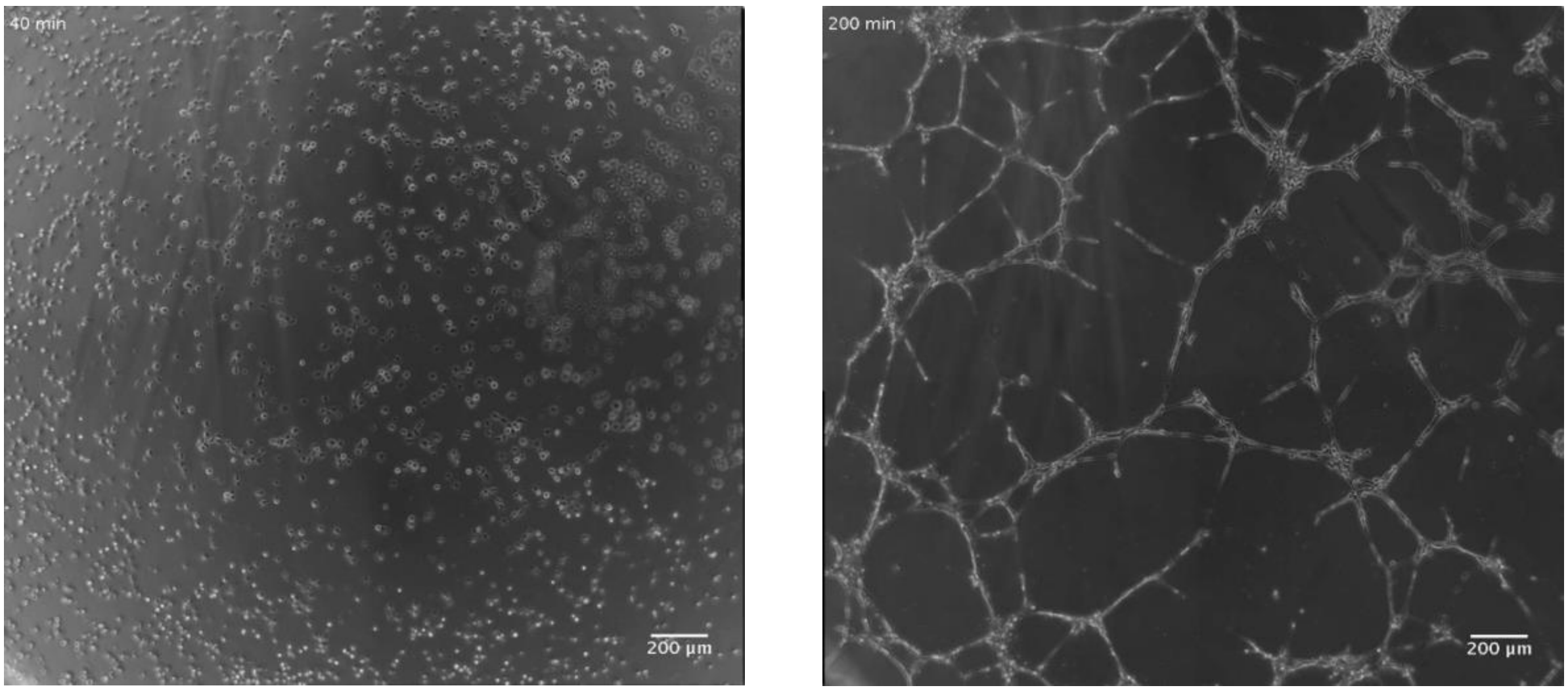

Video S2. Time-lapse video of HPMECs treated with ethanol vehicle control. Imaging conditions were identical to those in Video S1, with images taken every 20 minutes for 4 hours in a pre-equilibrated, humidified incubator chamber maintained at 37°C with 5% CO_2 and approximately 21% oxygen, using the same microscope and objective lens. Snapshot images show HPMECs at 40 minutes (left panel) and 4 hours (right panel) after EtOH vehicle treatment.

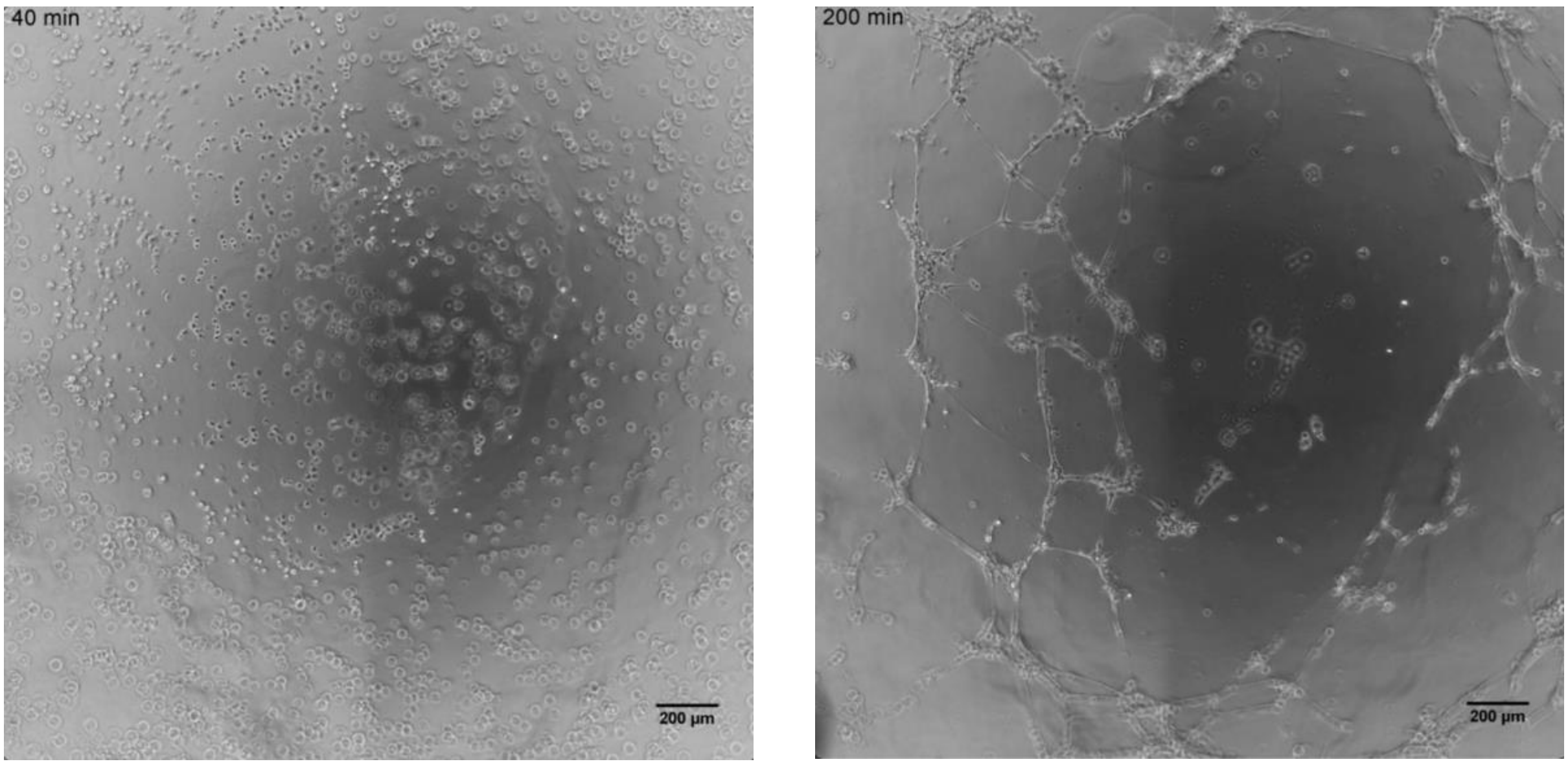

Tables S1-S6 are provided as a separate Microsoft Excel file.

Table S1. List of DEGs at 40 minutes post-treatment comparing EtOH control and RA-treated HPMECs.

Table S2. List of DEGs at 4 hours post-treatment comparing EtOH control and RA-treated HPMECs.

Table S3. The top 15 upregulated genes at 4 hours post-RA treatment. RARE (retinoic acid response element)-containing genes are highlighted in blue.

Table S4. The top 15 upregulated genes at 40 minutes post-RA treatment. No RARE-containing genes were identified.

Table S5. Evaluation of extracellular TGFα levels in the culture supernatants of HPMECs treated with EtOH control or RA. ELISA assay was performed using a Human TGF-alpha DuoSet ELISA kit.

Table S6. Detailed sample information for microarray profiling.

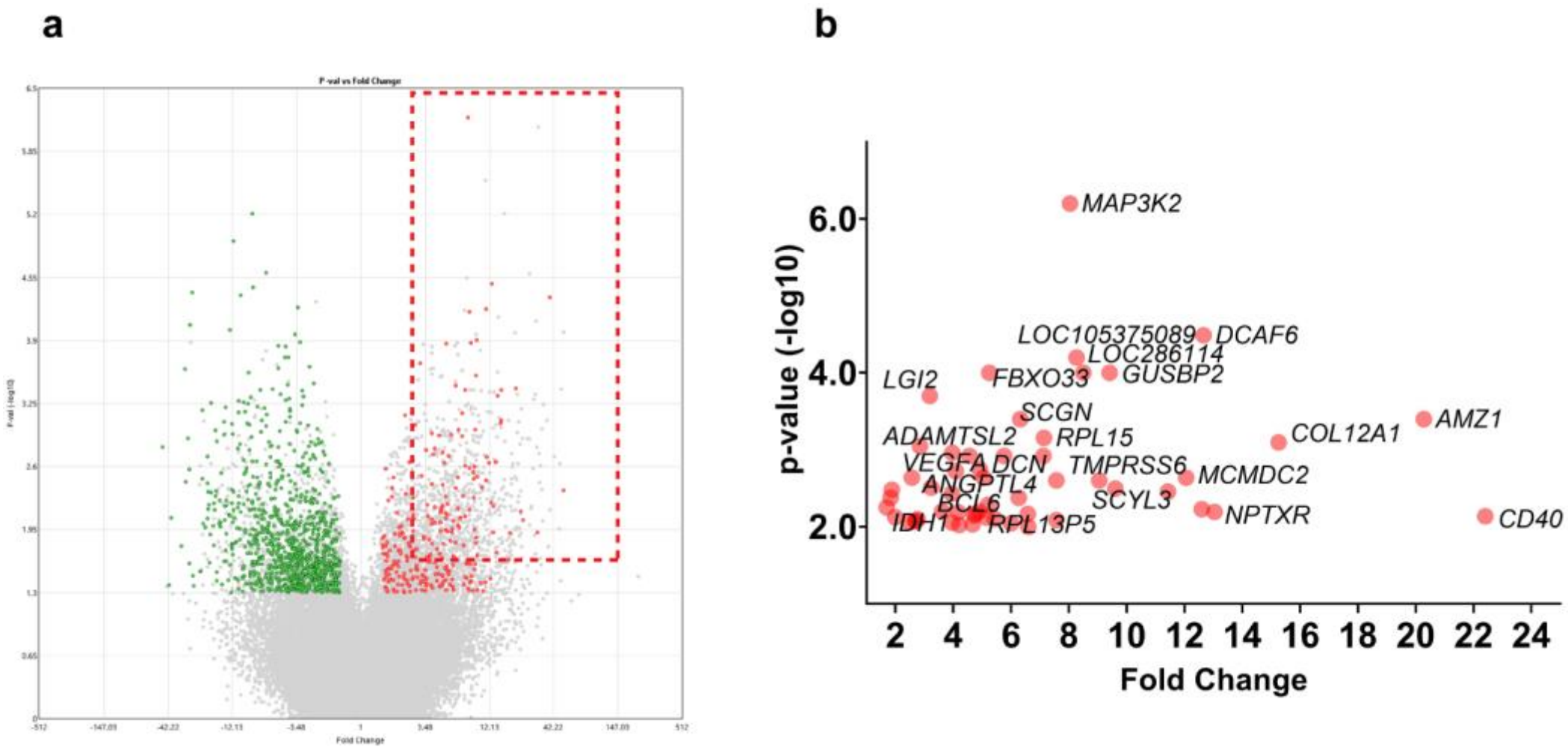

Figure S1. Differentially expressed genes (DEGs) in EtOH control versus RA-treated HPMECs at 40 minutes post-treatment. (a) Volcano plot showing DEGs between EtOH control and RA-treated HPMECs at 40 minutes post-treatment. Upregulated genes are shown in red; downregulated genes are shown in green. **(b)** Extrapolated volcano plot highlighting the top upregulated genes after 40 minutes of RA treatment.

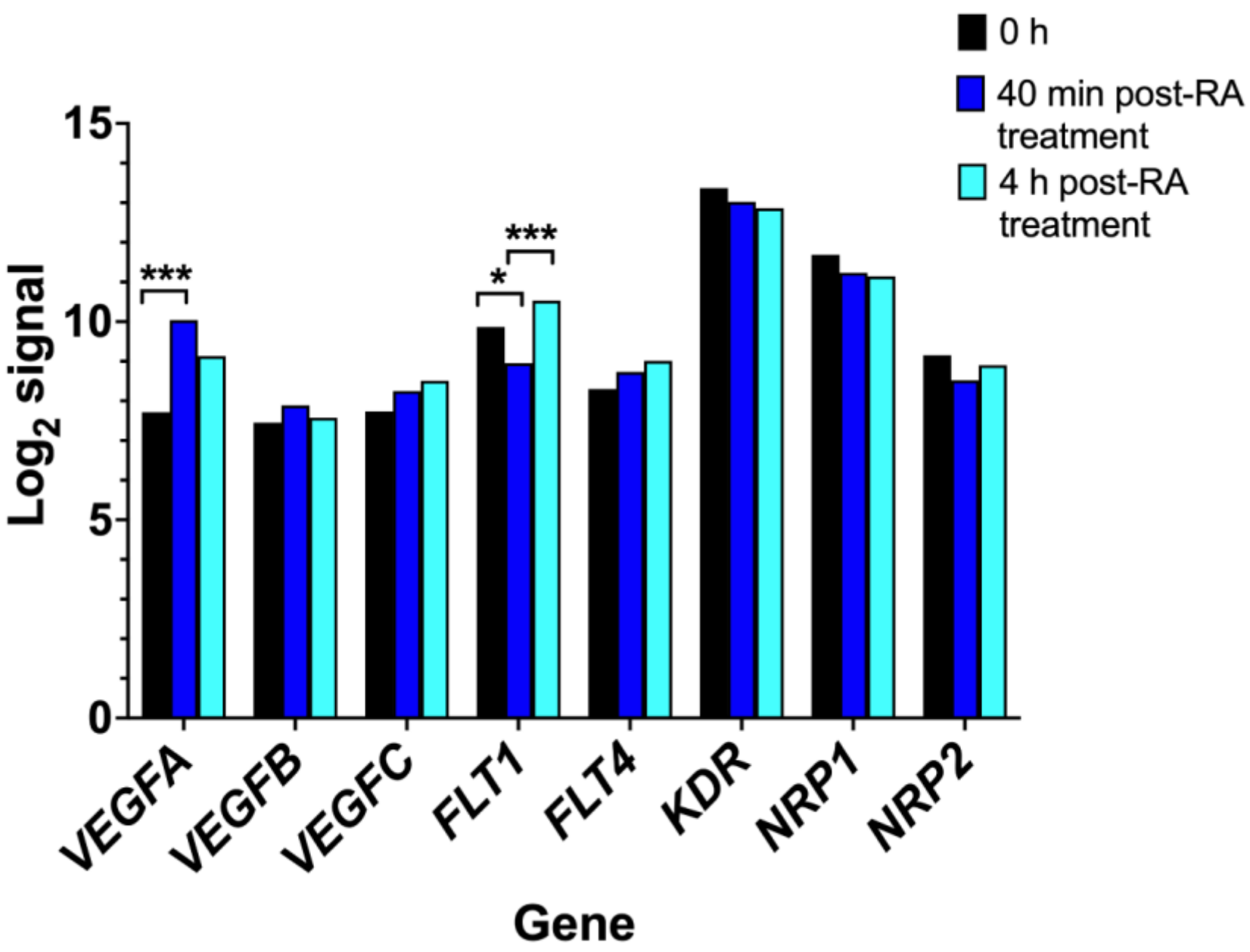

Figure S2. Upregulation of *VEGFA* and *FLT1* in HPMECs following RA treatment. Histogram showing expression levels of key genes in VEGF signalling, including *VEGFA, VEGFB, VEGFC*, *FLT1* (*VEGFR1), FLT4 (VEGFR3), KDR (VEGFR2), NRP1, and NRP2*.

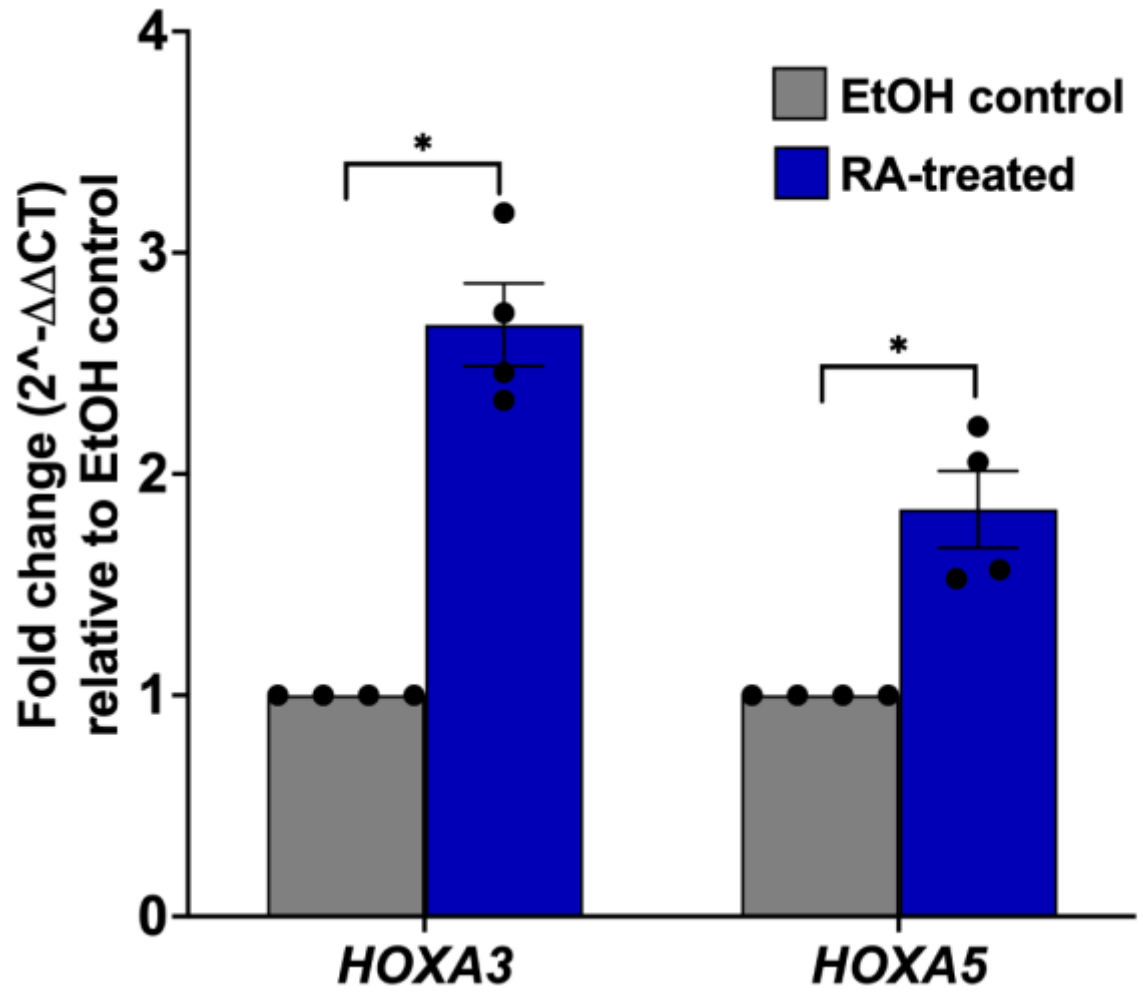

Figure S3. Upregulation of *HOXA3 and HOXA5* in RA-treated HPMECs. Histogram showing increased transcript levels of *HOXA3* and *HOXA5* at 4 hours post-RA treatment, assessed by qRT-PCR. HPMEC monolayers were cultured on fibronectin-coated plates. *HOXA3* and *HOXA5* were among the top upregulated genes identified in the microarray analysis. n = 4 independent experiments; each experiment was run in triplicate. Each dot represents the mean value per experiment. Data are presented as mean ± SEM; Mann–Whitney *U*-test, $*p < 0.05$.

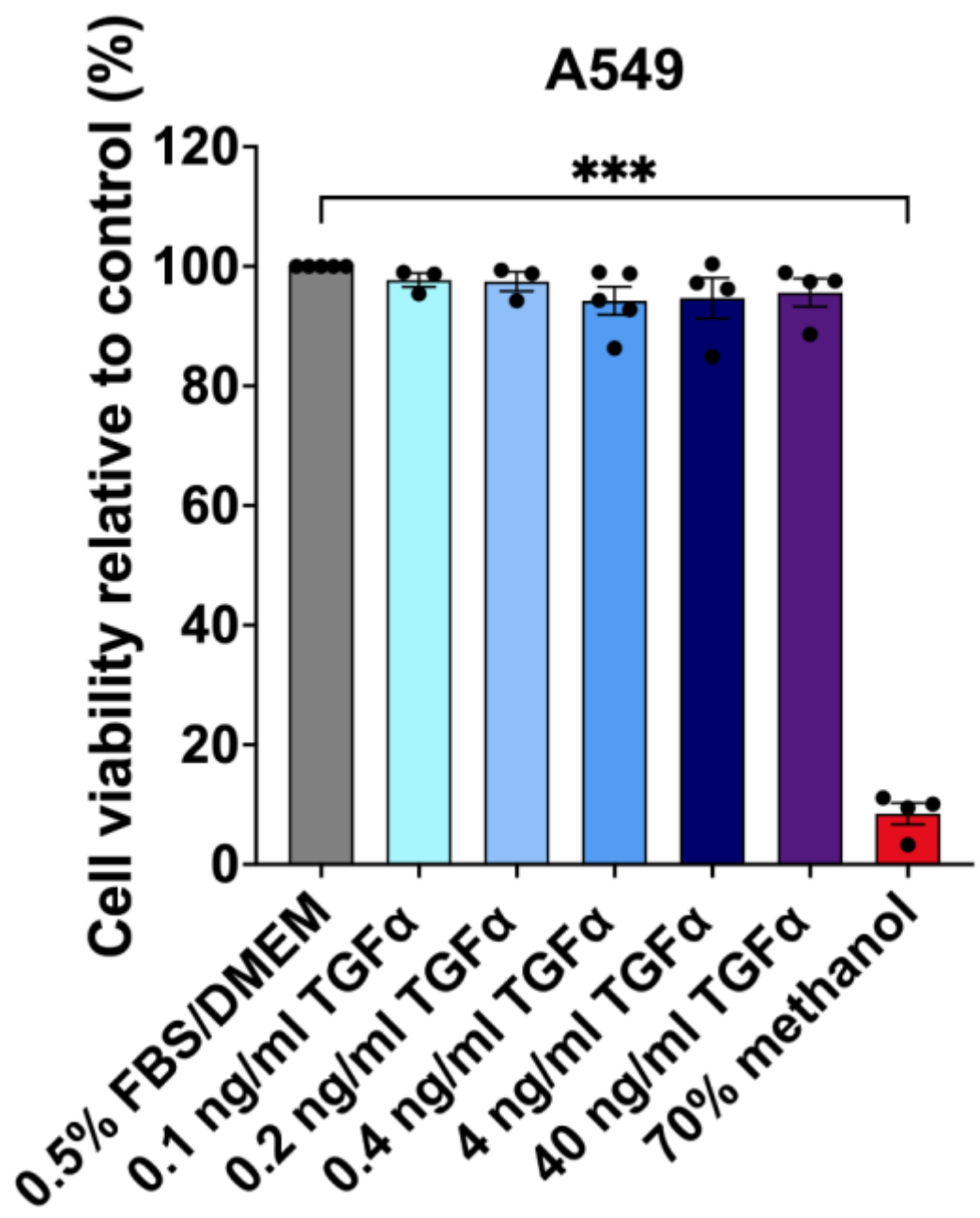

Figure S4. MTT metabolic assay results in A549 cells. Histogram showing cell viability in A549 cells treated with varying concentrations of TGFα. DMEM with 0.5% FBS served as a negative control, and 70% methanol-treated cells served as a positive control for cell death. Data are presented as mean ± SEM; Kruskal-Wallis with Dunn's multiple comparisons test, ***p < 0.001.

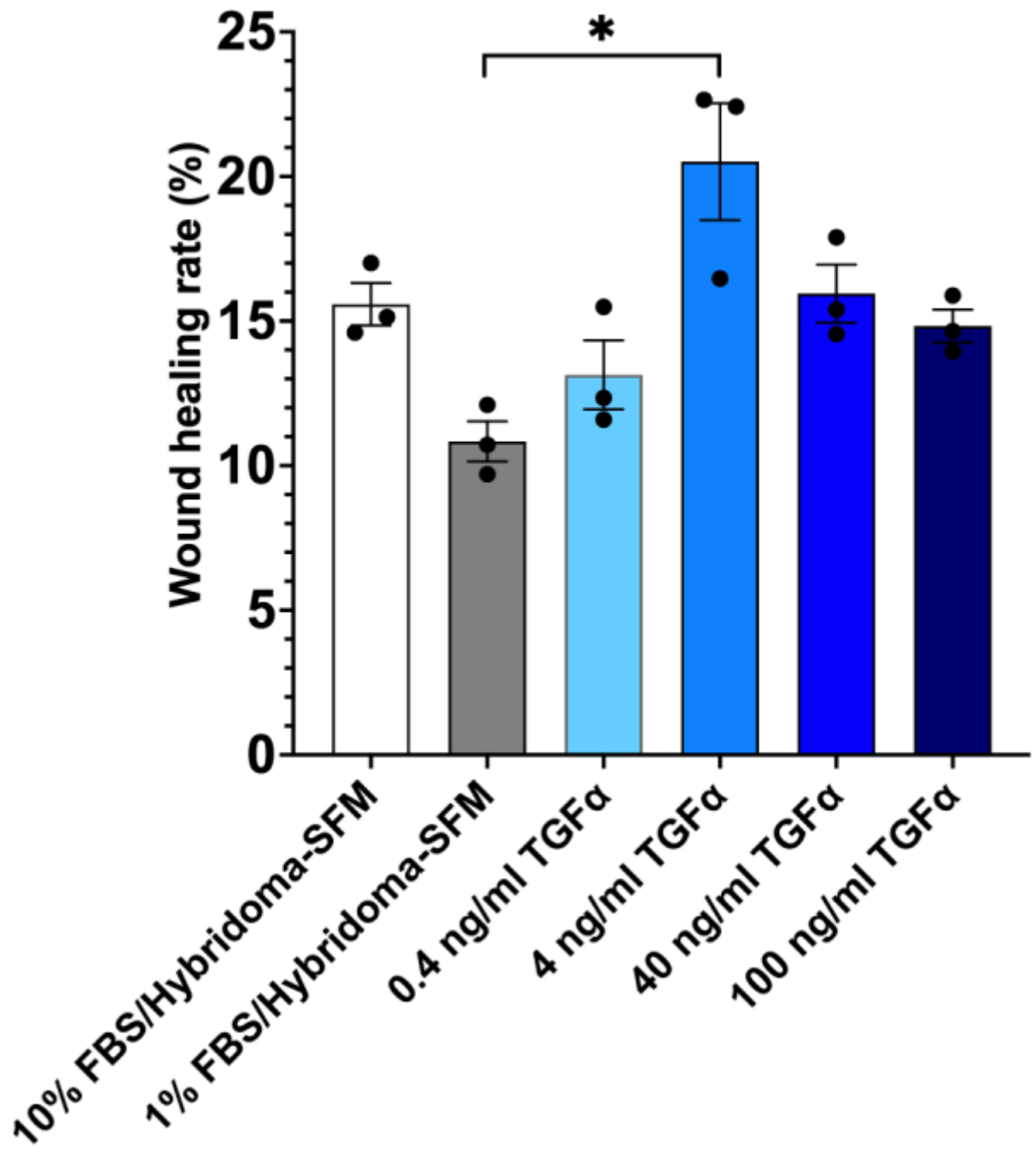

Figure S5. TGFα promotes wound healing in hAT2 cells in a dose-dependent manner. Histogram showing the percentage of wound healed at 24 hours post-scratch in hAT2 cells treated with hybridoma medium containing 10% FBS (positive control), hybridoma medium with 1% FBS (negative control), or varying concentrations of recombinant TGFα (0.4 ng/ml, 4 ng/ml, 40 ng/ml, and 200 ng/ml). n = hAT2 cells from 1 donor; three technical replicates per experiment; each dot represents a technical replicate. Data are presented as mean ± SEM; Kruskal-Wallis with Dunn's multiple comparisons test, $^*p < 0.05$.

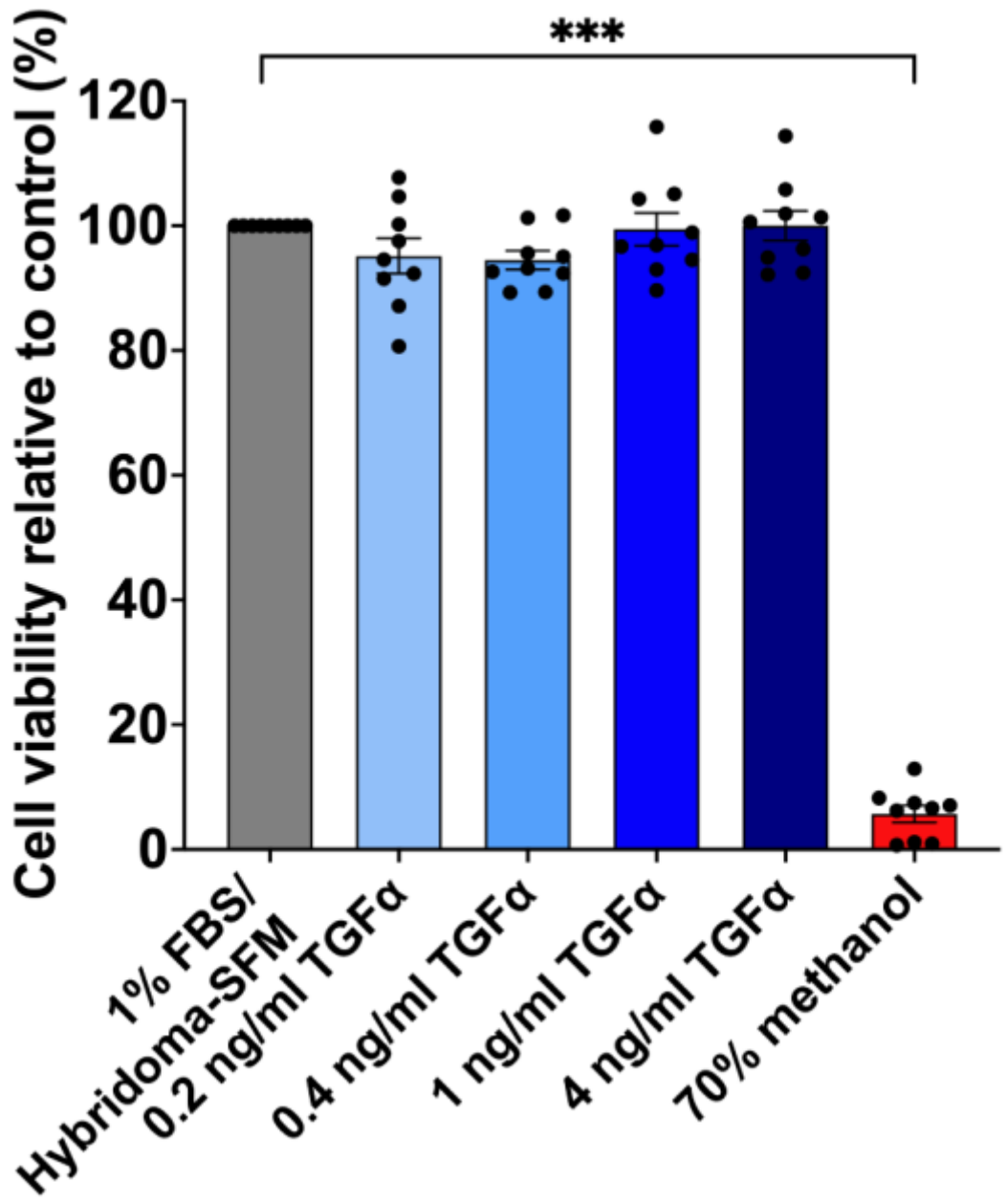

Figure S6. MTT cell viability assay in hAT2 cells. Histogram showing cell viability in hAT2 cells treated with different concentrations of TGFα (0.2 ng/ml, 0.4 ng/ml, 1 ng/ml, and 4 ng/ml). Hybridoma medium with 1% FBS served as a negative control, and 70% methanol-treated cells served as a positive control for cell death. *n* = hAT2 cells from 3 donors; three technical replicates per experiment; each dot represents a technical replicate. Data are presented as mean ± SEM; Kruskal-Wallis with Dunn's multiple comparisons test, ***$p < 0.001$.

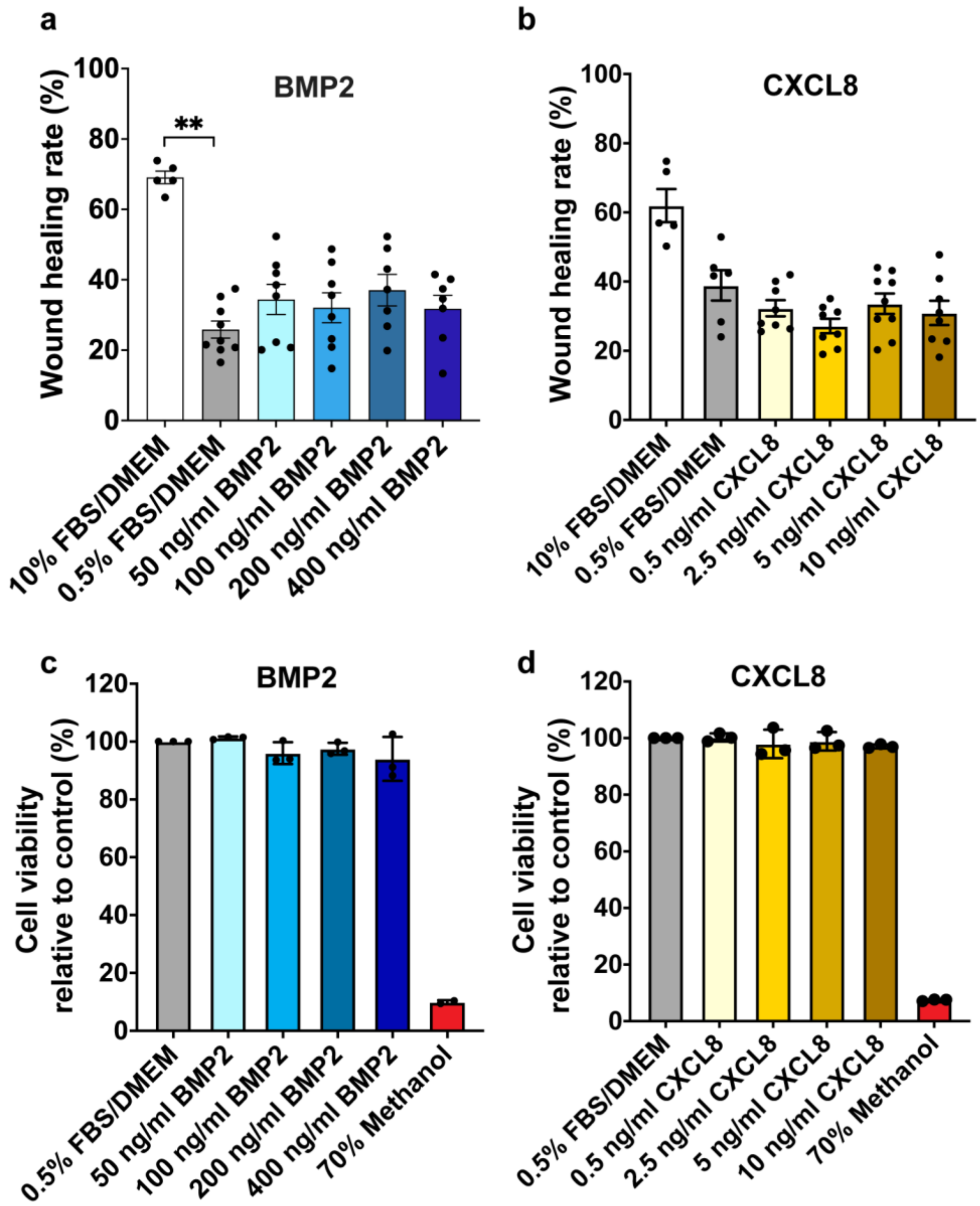

Figure S7. BMP2 and CXCL8 do not affect wound healing in A549 cells. (a-b) Percentage of wound healed at 24 hours in A549 cells treated with DMEM/10% FBS (positive control), DMEM/1% FBS (negative control), or varying concentrations of BMP2 (50 ng/ml, 100 ng/ml, 200 ng/ml, and 400 ng/ml BMP2) **(a),** and CXCL8 (0.5 ng/ml, 2.5 ng/ml, 5 ng/ml, 10 ng/ml CXCL8) **(b). (c-d)** Histograms showing cell viability in A549 cells treated with different concentrations of BMP2 and CXCL8. DMEM with 0.5% FBS served as a negative control, and 70% methanol-treated cells served as a positive control for cell death. n = three independent experiments; two or three technical replicates per experiment; each dot represents the mean value per experiment. Data are presented as mean ± SEM; Kruskal-Wallis with Dunn's multiple comparisons test, *$p < 0.01$.

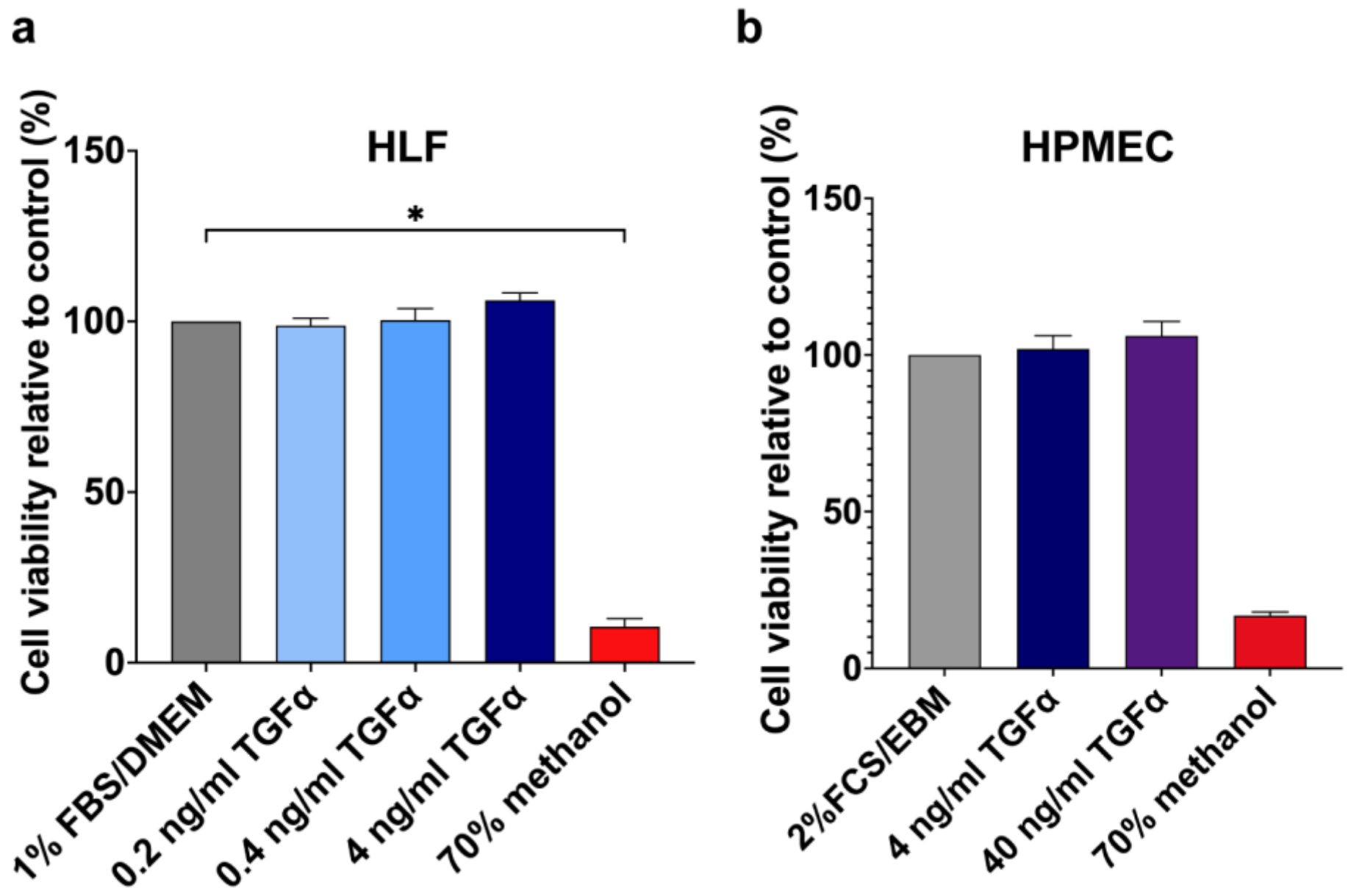

Figure S8. Assessment of cell viability in HLFs and HPMECs using MTT metabolic assays. (a-b) Cell viability in HLFs treated with TGFα at 0.2 ng/ml, 0.4 ng/ml, and 4 ng/ml **(a)** and HPMECs (4 ng/ml and 40 ng/ml) **(b)**. DMEM with 1% FBS and EBM with 2% FCS served as negative controls for HLFs and HPMECs, respectively, while 70% methanol-treated cells served as a positive control for cell death. For HLFs: n = HLFs from 3 donors; three technical replicates per experiment. For HPMECs: n = 1 experiment run in triplicate. Data are presented as mean ± SEM; Kruskal-Wallis with Dunn's multiple comparisons test was performed for HLFs, $^{***}p < 0.001$.

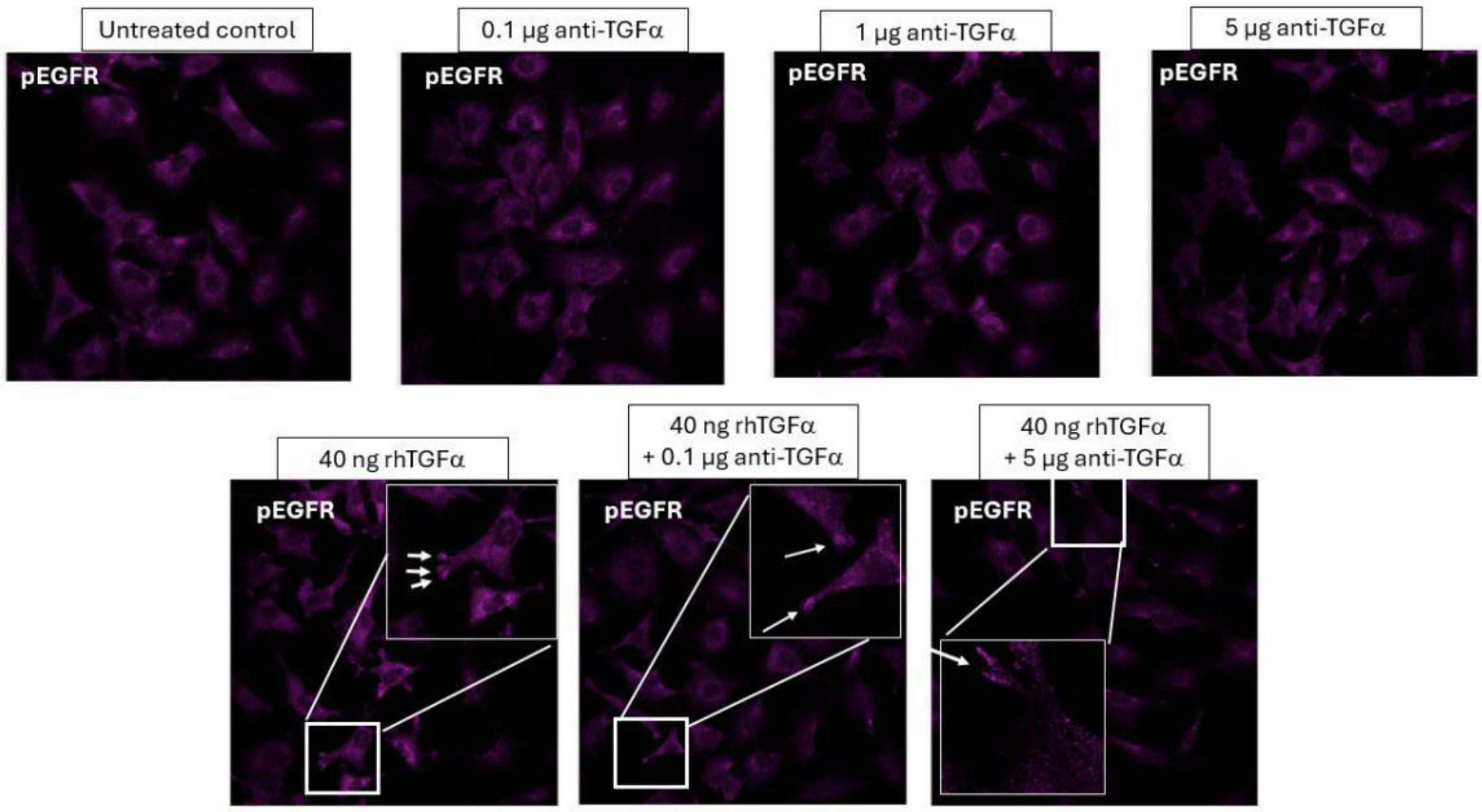

Figure S9. The upper panels show A549 cells either untreated or treated with varying concentrations of TGFα neutralising antibody, whereas the lower panels show A549 either treated with recombinant TGFα alone, or co-treated with recombinant TGFα and TGFα neutralising antibody. Cells were immunostained for phospho-EGFR (magenta). Images show that blockade of TGFα with neutralising antibody does not alter the distribution of phospho-EGFR in the cells compared to untreated A549 cells (upper panels). Treatment of A549 cells with recombinant TGFα leads to enriched membrane activation of EGFR as shown in the manuscript (lower left panel), and this distribution pattern does not change in cells co-treated with recombinant TGFα and TGFα neutralising antibody (lower middle and right panels).

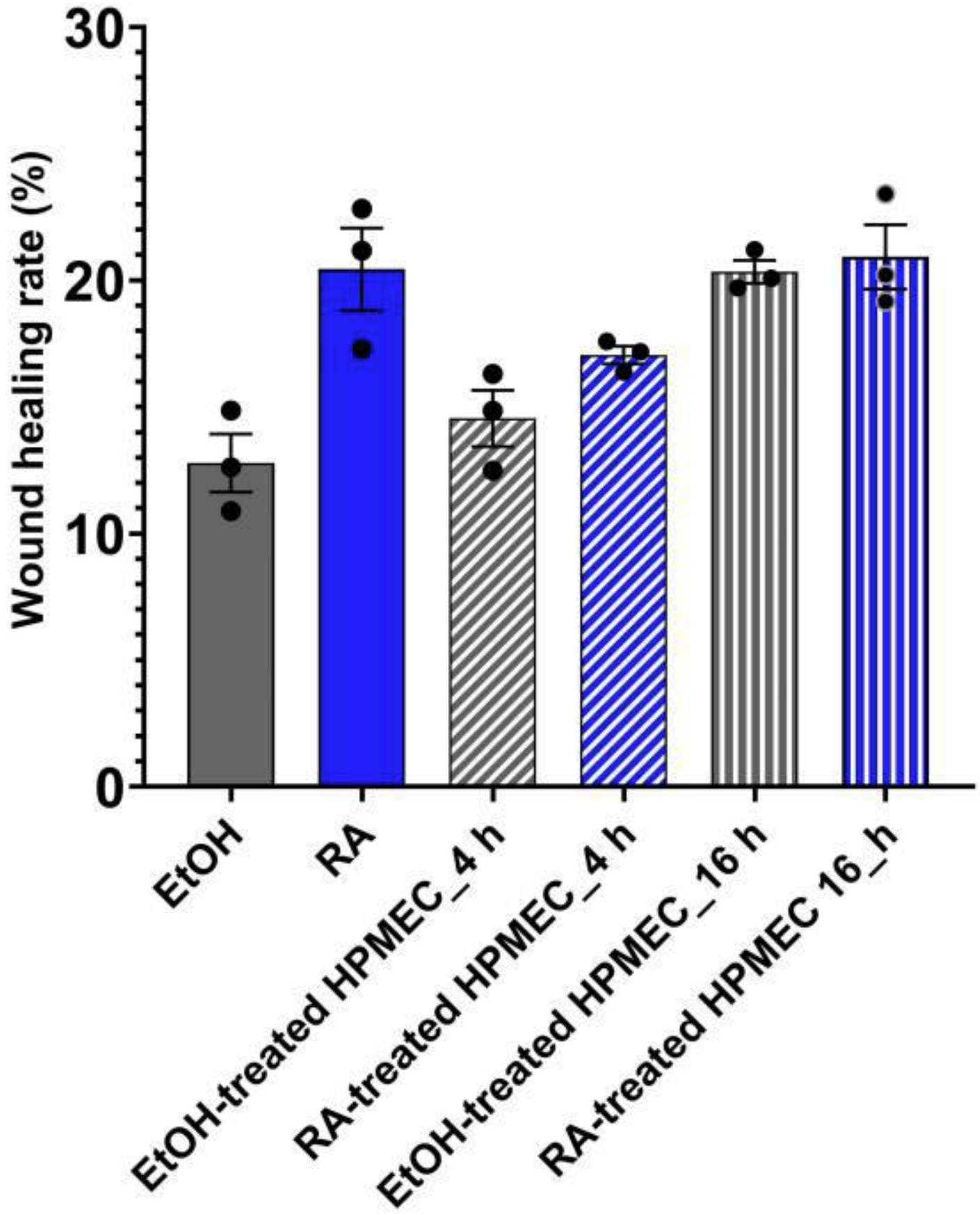

Figure S10. Application of EtOH- or RA-treated HPMEC conditioned media to scratch wound assays in A549 cells. Scratch wounded A549 cells directly treated with ETOH only (grey bar) or RA in ETOH (solid blue bar) were included as additional control groups.

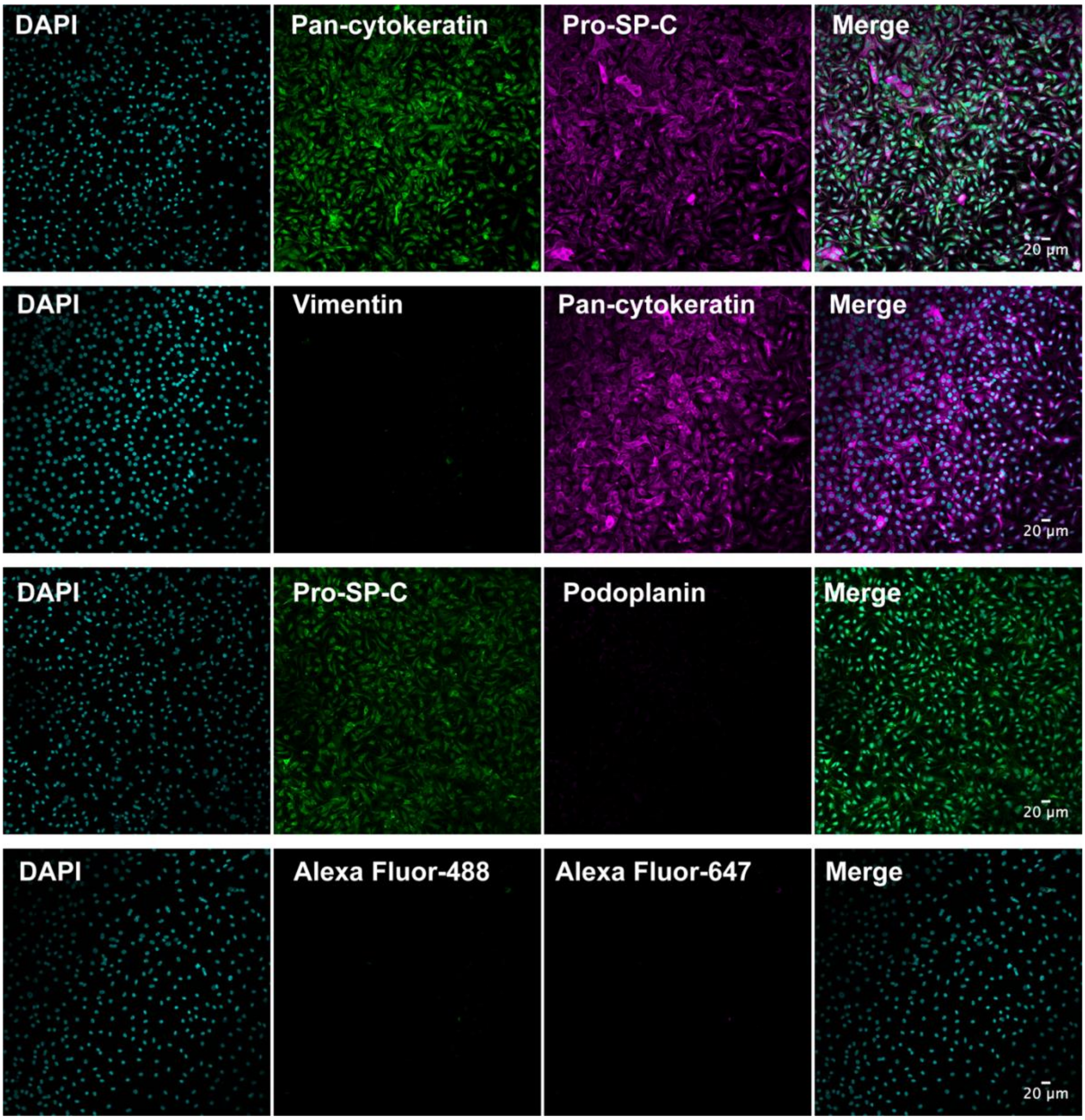

Figure S11. Immunostaining of hAT2 cells to confirm cell specificity. Representative images of hAT2 cells immunostained for pan-cytokeratin (a pan-epithelial marker), Pro-SPC (an AT2 cell marker), vimentin (a fibroblast marker), and podoplanin (PDPN; an AT1 cell marker) at 68 hours post-seeding. Negative controls lacking primary antibody are shown in the bottom panels. Nuclei were counterstained with DAPI (cyan). Images were captured using a Leica SP8 inverted confocal microscope with a HC PL APO 10×/0.40 air objective lens.
